# Detecting Multi-Scale Defects in Material Extrusion Additive Manufacturing of Fiber-Reinforced Thermoplastic Composites: A Review of Challenges and Advanced Non-Destructive Testing Techniques

**DOI:** 10.3390/polym16212986

**Published:** 2024-10-24

**Authors:** Demeke Abay Ashebir, Andreas Hendlmeier, Michelle Dunn, Reza Arablouei, Stepan V. Lomov, Adriano Di Pietro, Mostafa Nikzad

**Affiliations:** 1 School of Engineering, Swinburne University of Technology, Hawthorn, VIC 3122, Australia; 2 Aerostructures Innovation Research Hub (AIR Hub), Swinburne University of Technology, Hawthorn, VIC 3122, Australia; 3 School of Science, Computing and Engineering Technologies, Swinburne University of Technology, Hawthorn, VIC 3122, Australia; 4 Data61, Commonwealth Scientific and Industrial Research Organisation, Pullenvale, QLD 4069, Australia; 5 Department of Materials Engineering, KU Leuven, 3001 Leuven, Belgium

**Keywords:** manufacturing defects, non-destructive testing, continuous fiber-reinforced thermoplastics, machine learning, structural health monitoring, self-reporting

## Abstract

Additive manufacturing (AM) defects present significant challenges in fiber-reinforced thermoplastic composites (FRTPCs), directly impacting both their structural and non-structural performance. In structures produced through material extrusion-based AM, specifically fused filament fabrication (FFF), the layer-by-layer deposition can introduce defects such as porosity (up to 10–15% in some cases), delamination, voids, fiber misalignment, and incomplete fusion between layers. These defects compromise mechanical properties, leading to reduction of up to 30% in tensile strength and, in some cases, up to 20% in fatigue life, severely diminishing the composite’s overall performance and structural integrity. Conventional non-destructive testing (NDT) techniques often struggle to detect such multi-scale defects efficiently, especially when resolution, penetration depth, or material heterogeneity pose challenges. This review critically examines manufacturing defects in FRTPCs, classifying FFF-induced defects based on morphology, location, and size. Advanced NDT techniques, such as micro-computed tomography (micro-CT), which is capable of detecting voids smaller than 10 µm, and structural health monitoring (SHM) systems integrated with self-sensing fibers, are discussed. The role of machine-learning (ML) algorithms in enhancing the sensitivity and reliability of NDT methods is also highlighted, showing that ML integration can improve defect detection by up to 25–30% compared to traditional NDT techniques. Finally, the potential of self-reporting FRTPCs, equipped with continuous fibers for real-time defect detection and in situ SHM, is investigated. By integrating ML-enhanced NDT with self-reporting FRTPCs, the accuracy and efficiency of defect detection can be significantly improved, fostering broader adoption of AM in aerospace applications by enabling the production of more reliable, defect-minimized FRTPC components.

## 1. Introduction

Traditional composite manufacturing methods, including injection molding, solvent casting, stamping, and extrusion, are acknowledged for their effectiveness but are also recognized for their challenges [1,2,3]. These processes are often expensive and challenging to shape into complex forms [4,5]. They are time-consuming, require customized tools such as molds, and necessitate extra pre-processing and post-processing steps [6]. Recognizing these challenges emphasizes the need for enhanced and efficient composite fabrication methods across all sectors. The development of additive manufacturing (AM) of composites, such as three-dimensional (3D) printing, has emerged as a promising solution in intelligent manufacturing [3,7,8]. The AM technology produces accurate and complex structures [8]. For instance, Pal A. K. et al. [2] conducted a comparison of injection molding and 3D printing, highlighting key differences in cost, waste, and efficiency. Injection molding requires expensive, heavy molds and generates significant waste, estimated at around 96%, due to its subtractive nature. In contrast, 3D printing has lower fixed and operational costs, making it more cost-effective for small production runs and custom orders. It produces minimal waste, reducing it by up to 40% in metal applications and recycling approximately 95–98% of materials [9]. Additionally, 3D printing offers higher printing quality, greater flexibility for customized designs, and faster prototyping without the need for setup time, making it more efficient and adaptable than traditional manufacturing techniques. AM is the process of constructing an object layer by layer from a 3D CAD model, utilizing various raw materials and advanced techniques to ensure precision and customization, ultimately allowing for the creation of complex and highly detailed structures that would be challenging to achieve through traditional manufacturing methods [10,11]. A variety of techniques are included in AM technology, such as laminated object manufacturing (LOM) with plastic laminations, fused filament fabrication (FFF) with polymer filaments, stereolithography (STL) with photopolymer liquids, and selective laser sintering (SLS) with metal or plastic powders [12,13].

In the context of fiber-reinforced thermoplastic composites (FRTPCs), which consist of continuous or short fibers such as carbon, glass, or Kevlar embedded in thermoplastic resins like PEEK or PPS, AM offers the advantage of customized fiber orientations and controlled fiber-volume fractions. These fibers are classified into different types based on their composition, including aramid, glass, and carbon fibers, which contribute to varied mechanical and thermal properties. The thermoplastic matrices, such as PEEK, PET, and PA, offer design flexibility and recyclability, making them distinct from thermosetting resins, which are non-reversible after curing. Thermoplastic composites also present key advantages over thermosetting materials, including recyclability, faster processing, and repairability, making them increasingly attractive in industries like aerospace, automotive, and construction [4,14], especially with increasing concerns around end-of-life options for composite products. Unlike thermosetting composites, which exhibit higher stiffness and heat resistance, thermoplastic materials offer better impact resistance and repairability through reheating and remolding, making them ideal for high-performance applications and, at times, self-healing scenarios. Thermoplastic materials’ ability to be reheated and remolded offers superior design flexibility and sustainability compared to thermosets [15].

AM techniques can broadly be classified into seven main categories [16]. The first three—material extrusion, material jetting, and powder bed fusion—primarily use heat to achieve bonding. On the other hand, the remaining four methods—sheet lamination, binder jetting, material jetting, and vat photopolymerization—rely on chemical reactions for fusion during the production process [17]. Material extrusion-based additive manufacturing (MEAM) has developed progressively because of its ability to perform rapid printing and facilitate quick design iterations utilizing cost-effective materials [18]. During the last 20 years, this technology has substantially advanced. This technology is today regarded as a cutting-edge solution to various issues, such as extended design durations, limited fabrication flexibility, and exorbitant costs associated with conventional manufacturing methods [4,5]. Frketic et al. [19] conducted a review comparing the effectiveness of AM against conventional automated manufacturing techniques, such as automated tape laying (ATL), filament winding (FW), and automated fiber placement (AFP), in producing FRTPCs. The review highlights the considerable benefits of AM in accelerating product advancement and significantly reducing material wastage by 30 to 65%, emphasizing that the integration of continuous fibers into AM can yield mechanical properties comparable to those achieved in traditional composite processing [20,21,22,23]. AM processes provide the unique capability to tailor the strength of components by aligning fibers in specified orientations and controlling the fiber-volume fraction throughout the entire part [24,25]. Among additively manufactured composites, FRTPCs have become a promising option for producing high-performance parts in industries such as aerospace, automotive, and unmanned aerial systems (UAS) [25,26]. FRTPCs are also widely used in medical applications (e.g., prosthetics), sports equipment, and consumer electronics due to their lightweight, corrosion resistance, and durability under harsh environments. These materials offer improved mechanical, electrical, and thermal properties, ensuring their resilience under demanding conditions compared to neat plastic materials [27]. FRTPCs exhibit resistance to corrosion and high-temperature tolerance, rendering them suitable for challenging environments. Machine-learning (ML) techniques and their subsets, such as deep learning (DL) and convolutional neural networks (CNN), have shown great promise in enhancing structural health monitoring (SHM) systems [28]. ML models have the potential to predict damage progression and assess failure modes in real time, improving the accuracy of non-destructive testing (NDT) techniques. Combining SHM with ML facilitates early detection of defects, ensuring proactive maintenance strategies and extending the lifespan of critical composite structures [29]. Nevertheless, the AM of short fiber-reinforced thermoplastic composites (S-FRTPCs) introduces certain challenges, including printing defects and the need to strike a balanced trade-off between specific mechanical properties [19,25,26,30]. The 3D-printed S-FRTPCs exhibit notable performance enhancements over pure plastics. However, their mechanical properties remain significantly inferior to those of composites reinforced with continuous fibers. This discrepancy originates from the inherent superiority of fibers in specific stiffness and strength compared to the matrix material. Consequently, the design strategy leans towards weight-efficient engineering, with composite parts crafted to have fibers predominantly bearing and transmitting loads.

Substantial performance improvement is envisioned through the utilization of 3D printing specifically tailored for continuous fiber-reinforced thermoplastics, surpassing the capabilities of their short-fiber counterparts. However, the existing performance of 3D-printed continuous fiber-reinforced thermoplastic composites (C-FRTPCs) still falls short of conventionally processed composites. This shortfall can be attributed to imperfections such as voids, fiber misalignment, fiber breakage, matrix cracking, and inter- and intra-layer debonding resulting from poor adhesion between layers and between fibers and the matrix within the layers. Low-fiber-volume fractions can also contribute to the performance gap [25]. To unlock the full potential of these pioneering C-FRTPC structures, it is imperative to develop novel design, modeling, and analysis methodologies. Such types of innovations are crucial for identifying and optimizing the ideal applications of 3D-printed C-FRTPCs, addressing the current limitations, and paving the way for their broader and more effective utilization. When considering the strength, the crucial factors to consider are the length of the fibers within the component and their orientation [31]. In instances where the fibers are uninterrupted, uncrimped, uniformly dispersed, and aligned in the direction of applied stress, their ability to enhance the material is maximized. Conversely, the automation techniques for fabricating geometrically intricate components with fiber reinforcement, tailored to specific loads, remain inadequate [32,33].

Numerous review and original research articles have been dedicated to exploring the development of FRTPCs through 3D printing, focusing on both short and continuous fibers, with a significant emphasis on the macrostructural evaluation of mechanical, electrical, thermal, and structural characteristics [3,20,34]. However, there remains a gap in the research regarding the investigation of manufacturing defects in 3D-printed C-FRTPCs, particularly using MEAM methods with FFF materials. ML models, such as CNNs and DL algorithms, are increasingly integrated into the defect detection and classification process to improve precision in defect monitoring. These AI-driven approaches allow for the real-time identification of voids, cracks, and delamination in FRTPCs, enhancing the efficiency and reliability of NDT methods [35]. FRTPCs play a pivotal role in advancing the field of SHM for aerostructures due to their exceptional structural and non-structural attributes, including self-reporting, self-sensing, and self-monitoring capabilities, as well as their resilience to harsh environmental conditions [36]. This makes them an ideal choice for aerostructural components, especially when subjected to demanding applications like fire-exposed scenarios, extreme heat, corrosive environments, and salt exposure. The fusion of SHM with ML algorithms is transforming the field by enabling predictive maintenance and reducing downtime in aerospace and other critical applications [37,38]. Through such integrations, SHM systems can assess damage accumulation and provide timely alerts for repair, improving overall safety and operational efficiency.

This review looks at the study of AM defects in FRTPCs, explicitly focusing on determining the effect of FFF-induced multi-scale defects on FRTPCs. The review covers a lot of ground, such as manufacturing defects in both short and continuous FRTPCs, how AM defects are classified and formed, how to describe FFF-induced multi-scale defects particularly small-scale (below 100 μm) defects, and both basic and advanced NDT methods for finding multi-scale defects, subsequently leading to improvements in in situ SHM techniques. It delves into hybrid approaches integrating NDT with ML algorithms to gain deeper insights into AM defects in FRTPCs. The review concludes by addressing identified research gaps, offering future perspectives, and providing recommendations for further advancement in the field. Key areas of future research include the development of more efficient ML models tailored for composite defect detection and enhanced SHM frameworks capable of processing large datasets from multiple NDT sources in real time.

## 2. Manufacturing Defects in FRTPCs

### 2.1. Introduction

**Figure 1:**
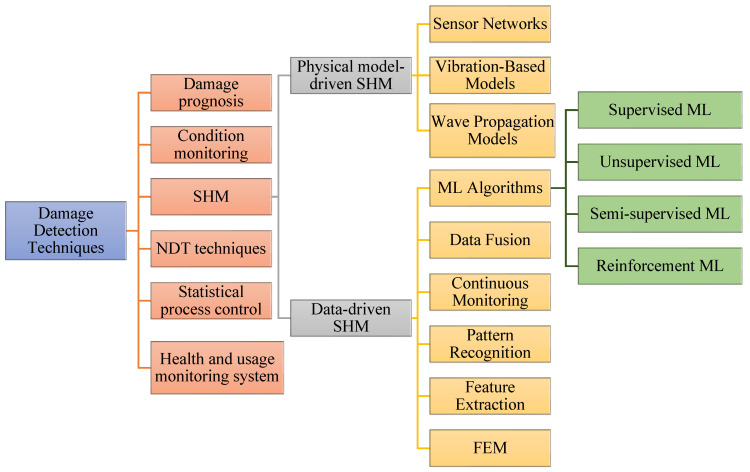
Broad classification of damage-detection techniques and SHM [29].

FRTPC materials play a pivotal role across diverse domains, from automotive and aerospace, including aerospace, aircraft, and UAS structures, to sectors like medical and maritime infrastructures [32,39,40]. They hold a significant position in the global economy, contributing indispensably to the daily lives of people worldwide. Nevertheless, these invaluable resources frequently undergo premature deterioration as they approach the end of their operational lifespan, grappling with flaws emerging during the production process. The impending challenges, combined with defects in the production procedures, are notably significant. These defects present a substantial failure of the mechanical properties of FRTPCs, diverting them from their originally intended specifications. Unlike damages occurring after loading, such as matrix cracks and delamination, manufacturing defects materialize as anomalies instigating alterations in composite characteristics [41]. The urgency of tackling these obstacles cannot be emphasized enough, considering that substituting structures affected by manufacturing defects involves considerable expenditures and laborious endeavors, and it imposes a strain on existing financial and human resources. Acknowledging the economic and societal influences of potential breakdowns, engineers have continuously undertaken to formulate various methods to enhance the safety and structural reliability of these structures [7,29]. Figure 1 provides a graphical depiction of the diverse disciplines employed in SHM for identifying damages.

**Figure 2:**
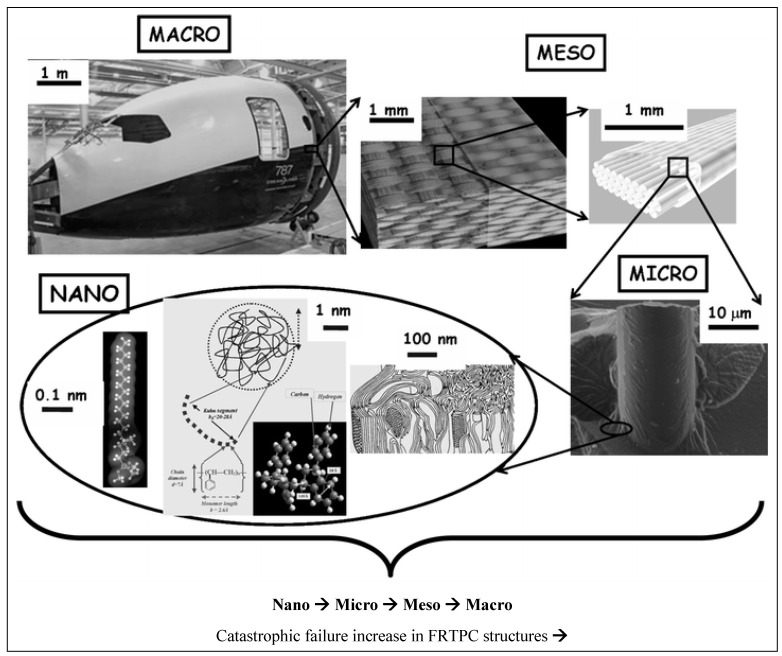
Structural integrity influenced across multiple scales, ranging from the nano to micro, meso, and macro levels. Reproduced with permission from [56], © 2008 Springer Nature.

Manufacturing defects are the primary factors causing variations in the mechanical properties of FRTPCs from their intended specifications [42]. These defects are faults that lead to variations in composite properties, distinct from damage that arises only after the composite undergoes loading, such as matrix cracks and delamination. Some studies [43,44,45] describe another type of anomaly in FRTPCs, referred to as “design features”, which arise from unavoidable micro- to meso-scale structures due to the geometric aspects of the component, such as fiber misalignments or distortions of the tow at sharp bends and corners. Determining whether a manufacturing flaw can be corrected involves assessing if adjusting processing parameters can eliminate it. Defects in composites are classified as fiber-, matrix-, or interface-related. Fiber issues include waviness, misalignment, and breakage, while interface defects involve debonding and delamination. Matrix defects often consist of incomplete curing and voids, which are critical as they significantly impact composite performance and failure mechanisms, and they frequently occur in manufacturing processes. Consequently, voids have been widely studied as a manufacturing defect [42,46,47,48,49,50,51]. While void content has been recognized as a parameter-affecting mechanical property, in-depth analysis of void effects requires consideration of their characteristics, such as shape, size, and location [42,50,52]. Understanding void effects requires evaluating these characteristics in correspondence with their formation [42]. Voids form, evolve, and are assessed differently during FRC processing through various manufacturing techniques and technologies [42,53,54]. These differences arise from variations in the thermodynamic and rheological phenomena specific to each processing method. For example, in Liquid Composite Molding (LCM), significant research has been dedicated to understanding the void formation and its evolution, while autoclave curing predominantly evaluates the void content in final components [42]. Innovative methods such as OoA (out-of-autoclave) processing, automated prepreg placement, and AM have driven studies on void formation in FRTPCs across micro, meso, and macro scales. Micro voids develop within fiber tows, with meso voids between them and macro voids in larger visible sections of the preform. The formation of micro and meso voids is influenced by localized flow at the micro scale, while macro voids stem from overall flow patterns treating the preform as a uniform structure. These flows are strongly interconnected, affecting overall void formation [55]. Figure 2 demonstrates the impact on structural integrity across multiple size levels, ranging from nano to micro to meso to macro scales [56]. This graphical representation underscores the interconnectedness of structural challenges and defects across these diverse size dimensions within the composite material.

### 2.2. Multi-Scale Defects

**Figure 3:**
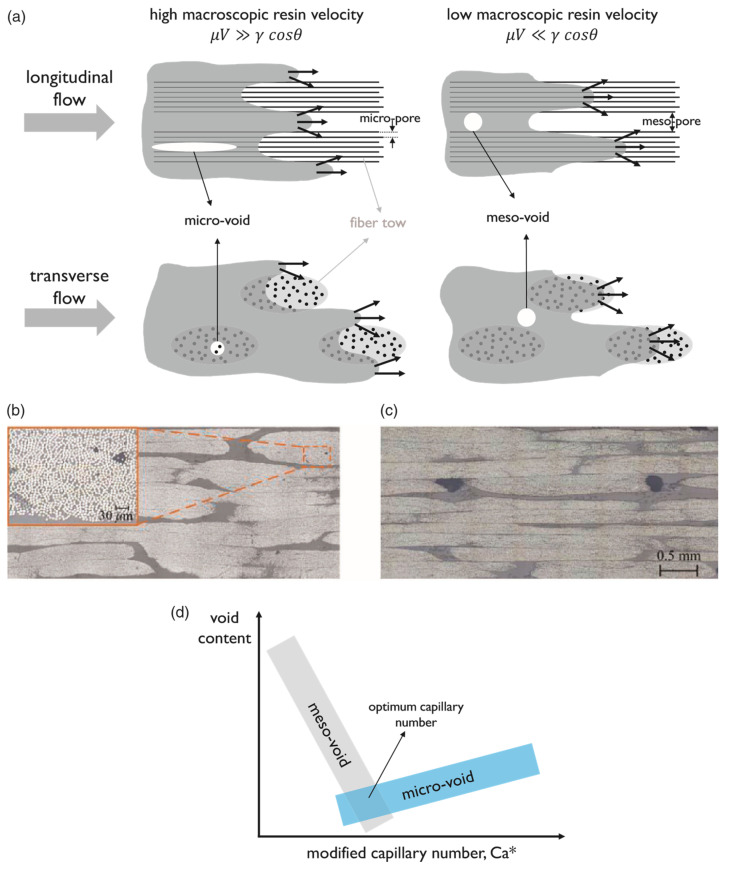
(**a**) Diagram illustrating void formation during longitudinal and transverse flow in liquid composite molding of a dual-scale fibrous preform, demonstrating the interaction between viscous and capillary flows; inclined arrows represent transverse impregnation of the tow. Micrographs display (**b**) micro voids within fiber tows and (**c**) meso voids between tows. (**d**) Schematic depicting the relationship between void content and the modified capillary number, indicating the optimal capillary number for reducing void formation. Reproduced with permission from [42], © 2019 SAGE Publications.

**Figure 4:**
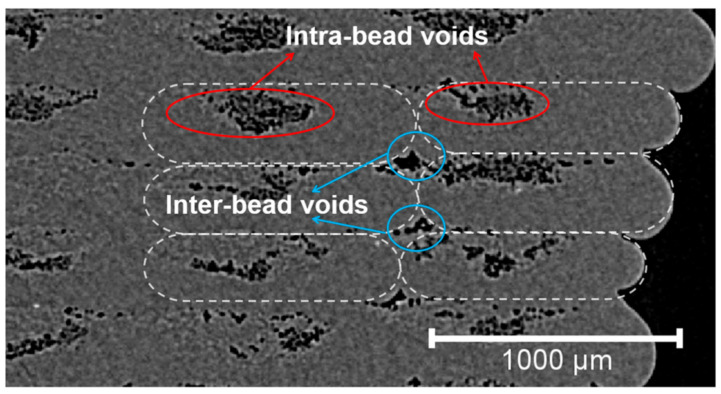
A typical Micro-CT image of FFF-produced CFRTPC sample containing different types of voids [65]. Reproduced with permission under CC BY 4.0, © 2023 MDPI.

**Figure 5:**
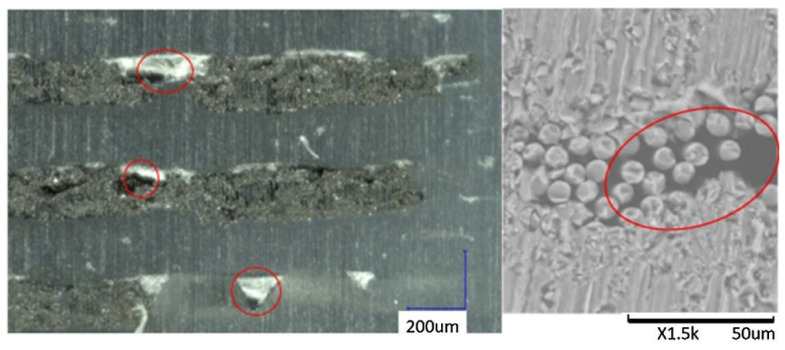
Air inclusion within a carbon fiber bundle was observed through optical microscopy (**left**) and electron microscopy (**right**). Reproduced with permission from [66], © 2017 Elsevier.

In the realm of conventional polymer composite manufacturing, resin-transfer molding (RTM) has emerged as a prominent technique for producing carbon fiber-reinforced composites (CFRCs), boasting advantages such as versatility and cost-effectiveness [42]. However, the production of high-quality CFRCs is not without its challenges, with manufacturing defects like macro voids representing a pertinent concern. Macro voids are distinct regions within CFRCs where the resin, crucial for binding continuous fibers, has failed to infiltrate effectively during the RTM process. The growth of these macro voids can be due to various factors, including irregular permeability of the preform material, which can disrupt the uniform flow of resin, resulting in voids (Figure 3,Figure 4 and Figure 5). Improperly positioned injection points for resin can lead to uneven resin distribution throughout the CFRC, exacerbating the issue of macro voids. Additionally, inserts, ribs, and cores in the mold configuration can impede resin flow, leading to the formation of macro voids.

Macro voids within CFRCs can be categorized based on their scales. Firstly, macro-scale voids are those that are readily visible to the naked eye, typically substantial in size and indicative of significant resin flow irregularities. Conversely, meso-scale voids are smaller and may necessitate closer scrutiny, often requiring microscopic examination to be identified accurately. Micro-scale voids, the most diminutive of the macro voids, can only be observed under high magnification and are often linked to intricate resin flow patterns (Figure 3). These voids manifest in various locations within CFRCs, with common occurrences found near insertion points where continuous fibers are initially integrated into the process. Challenges arise here due to the difficulty of uniformly saturating the fibers at these entry points. Additionally, macro voids tend to form in regions associated with resin injections, especially when irregularities surface due to inappropriate placement or design. Areas featuring complex geometric characteristics, such as corners or overhangs, also present a propensity for macro voids as disrupted resin flow patterns can emerge. Effectively addressing macro voids necessitates a comprehensive understanding of their formation mechanisms. Strategies to mitigate these manufacturing defects often center around optimizing resin flow control, refining injection point placement, and enhancing mold design. By proactively addressing these factors, it becomes possible to curtail the incidence of macro voids, thereby elevating the overall quality of CFRC components generated through RTM. Ongoing research endeavors and innovative approaches continually propel the quest to confront this challenge and advance the potential of RTM in the realm of CFRC manufacturing [57].

### 2.3. Defects in Additively Manufactured FRTPCs

The most common manufacturing defects in 3D-printed carbon fiber-reinforced thermoplastic composites (CFRTPCs) include voids, fiber misalignment, fiber breakage, and delamination. Voids are frequently observed due to poor resin impregnation in fiber bundles, which occurs when the resin is not adequately molten during the printing process, leading to excessive void content in the thermoplastic matrix [58]. Fiber misalignment and breakage frequently occur due to torsional deformation during the steering path and excessive forces exerted by the nozzle, particularly at sharp turning angles and small curvature radii. These factors contribute to shape inaccuracies and fiber twisting [41,59]. Other common defects, such as delamination, gaps, and non-uniform tape consolidation, often result from tension build-up caused by the movement of the print head relative to the laminated tape, leading to tape warping and narrowing below its intended width [60]. Moreover, redundant material accumulation, scratching, warping, and matrix buckling are frequently observed defects, with matrix and interface failures being the dominant failure mechanisms under bending stresses [61,62]. The presence of such defects can significantly degrade the mechanical properties of the printed composites, including tensile strength and fatigue resistance. Consequently, techniques like laser ultrasonic testing (LUT) and various NDT methods are utilized to detect and mitigate these defects [7,63]. The aligned fiber deposition (AFD) technique has demonstrated effectiveness in reducing fiber waviness and twisting, promoting smoother filament deformation and decreasing air voids and fiber breakage during printing [59]. Furthermore, optimizing factors such as orientation and tape raster offset can reduce defects like delamination and void formation, improving the overall quality of the printed composites [60]. The integration of complementary detection methods and process optimization is vital for enhancing both manufacturing efficiency and the structural performance of 3D-printed CFRTPCs [7,62,63]. In a study by Zhang et al. [64], a 3D printing extruder was developed utilizing in situ impregnation for C-FRTPCs. This approach aimed to reduce void content while maintaining high mechanical performance. Inadequate resin impregnation and insufficient consolidation pressure often lead to porosity in printed composite structures. These voids can be categorized into two main types: intra-bead voids (red circles in Figure 4), which occur within fiber bundles due to incomplete resin impregnation, and inter-bead voids, which are found between printed filaments due to poor filament flow caused by a cold interface and insufficient pressure. Each type of void arises from distinct manufacturing issues, affecting the structural integrity of the composite. For instance, air bubble (voids) and inclusions within the carbon fiber bundle were observed through optical microscopy (left) and electron microscopy (right), as illustrated with red circles in Figure 5.

**Figure 6:**
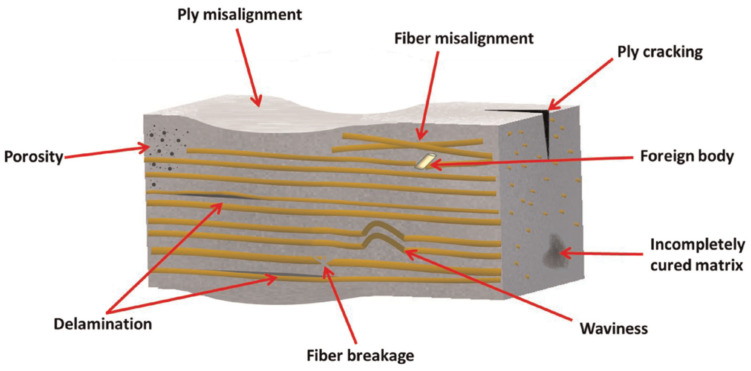
Manufacturing defects in composite structures. Reproduced with permission from [68], © 2024 SAGE Publications.

Regardless of the processing technique used, introducing defects into polymers and FRTPCs is inevitable throughout the process of manufacture. Undoubtedly, since no structure can be completely devoid of imperfections, achieving a material structure without defects involves establishing a minimal threshold for quantifiable defects [67]. Defects in polymer composites may be attributed to several sources, including insufficient curing and voids in the matrix, misaligned or cracked fibers, and abnormalities in filler distribution within the composite. Additionally, defects can also arise through delamination at the interface between various components of the composite. In the realm of the 3D printing of FRTPCs, it is vital to have a deep understanding of the different types of defects, the factors leading to their formation during the printing process, and an accurate assessment of their impact on key material properties. This knowledge is vital for effectively controlling, optimizing, and maximizing the potential of the 3D printing process. Composites are prone to various manufacturing defects, some of which are specific to particular techniques [68]. Analyzing the circumstances that lead to defects is crucial. The most frequent manufacturing defects are introduced in Figure 6 as follows:

Thermal 3D printing, such as SLS and FFF, inherently introduces meso–micro-scale heterogeneities, including voids, into the printed components due to temperature variations [53]. Voids may arise in the context of FFF due to variations in filament diameters, the presence of air trapped inside the material matrix, or gaps that exist between individual beads and layers. These defects may significantly impact the mechanical characteristics of the final components, underscoring the need to understand their effect. The dimensions, configuration, and arrangement of empty spaces and cavities are contingent upon the properties of the material and the variables involved in the fabrication process. The presence of porosity within the structure of composites results in the development of internal stresses, leading to a significant decrease in their mechanical properties [68]. The properties include interlaminar shear strength, bending strength and modulus in both longitudinal and transverse directions, and tensile strength and modulus in both orientations, as well as compressive strength and modulus [42]. Such degradation of mechanical properties can notably impact the overall service life of these materials in practical applications, undermining their durability and performance. Therefore, it is crucial to comprehensively comprehend the intricate relationship among these aspects to attain the intended performance of the items.

### 2.4. Effect of Manufacturing Defects on Performance of FRTPCs

**Figure 7:**
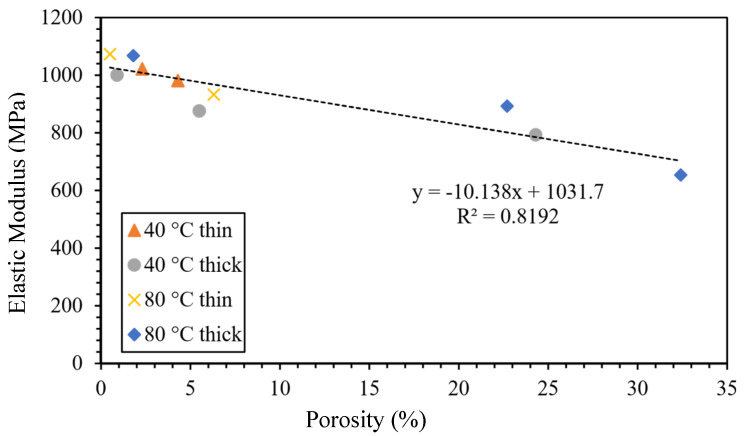
The relationship between porosity and the elastic modulus of 3D-printed parts [69]. Reproduced with permission under CC BY 4.0, © 2019 MDPI.

Liao et al. [69] conducted a comprehensive study on the influence of porosity on the mechanical attributes of PLA produced via FFF. They investigated the effects of bed/platform temperatures (40 °C and 80 °C) along with layer-thickness variations, including thin samples with 4 layers and thick samples with 24 layers. Interestingly, the thin samples exhibited comparable internal morphology to their thicker counterparts, both featuring small triangular voids. Regardless of the sample type, the upper and middle layers consistently showed higher porosity levels compared to the lower layers, indicating the impact of layer positioning on void formation. A key outcome of their study was the identification of a clear linear relationship between porosity and the elastic modulus, as illustrated in Figure 7. While fibers are generally the dominant contributors to the elastic modulus in fiber-reinforced composites, voids within the matrix can play a crucial role in diminishing the mechanical performance of the structure. As void content increases, the effective load transfer between fibers and the matrix decreases, as voids act as stress concentrators that weaken the bonding and interfacial strength between the fibers and the matrix. This disruption of matrix continuity promotes the early onset of crack initiation and accelerates crack propagation under mechanical loads, leading to a reduction in stiffness and structural integrity. Moreover, porosity compromises fiber–matrix adhesion, reducing the composite’s ability to resist deformation. Increased porosity also limits the matrix’s ability to maintain fiber alignment, a critical factor in load-bearing applications. As Liao et al.‘s findings suggest, the elastic modulus of FFF-produced PLA decreases linearly with increasing porosity, underscoring the importance of controlling void formation during the printing process. This insight highlights that even in cases where fibers dominate the composite’s mechanical properties, the matrix’s voids play a significant role in reducing overall performance by undermining load-transfer efficiency and promoting failure mechanisms. Thus, precise control over porosity formation during the fabrication process is essential to ensure the mechanical performance, dimensional stability, and durability of FFF-printed components.

Wang et al. [70] conducted experiments and micromechanical modeling to examine the impacts of micro pores on the characteristics of FFF-produced material. The X-ray CT (XCT) technique was utilized to quantitatively analyze the intricate 3D microscopic characteristics of the internal pores, encompassing their dimensions, morphology, distribution, and spatial arrangement. Subsequently, experimental investigations were conducted to evaluate the mechanical properties of the material. A micromechanical model was developed to estimate these properties by incorporating the microscopic characteristics of pores, as identified through XCT analysis. The model defines porosity as the ratio of the total pore volume to the total material volume. These discontinuities, introduced by pores, act as stress concentrators, disrupting the load transfer between fibers and the matrix and resulting in the degradation of fiber–matrix bonding and the overall performance of the composite structure. This micromechanical approach allows designers to predict the elastic properties of 3D-printed materials based on porosity data derived from XCT results. This presents an opportunity to reduce expenses associated with destructive testing. Similar research findings by Gordeev et al. [71] have shown that products manufactured using FFF exhibit poor performance due to high structural defects, such as porosity (summarized in Table 1 and Table 2). Porosity impacts not only the overall strength of the part but also affects the adhesion between layers, leading to increased anisotropy and greater susceptibility to crack propagation. These limitations impede the practical use of FFF in functional prototype development and the direct fabrication of products exposed to gases and liquids. The study introduced a simple and efficient method for evaluating the quality of 3D-printed products. The findings of this study indicate a strong correlation between the geometric arrangement of a printed product and its permeability, with a decrease in permeability seen as the shape transitions from a cylinder to a cube, pyramid, sphere, and, finally, a cone. The major determinants of 3D-printing quality were identified by the authors as wall geometry and structure, as well as the filament feed rate described by G-code. Optimizing these process parameters helps to reduce porosity levels, improving layer bonding, dimensional stability, and mechanical strength. Through the enhancement of these parameters, there was a notable improvement in both the quality and sealing aspects. The primary outcome of the study revealed that traditional printers and filament materials have the capability to generate 3D-printed things of exceptional quality. Zhang et al. [72] investigated how specific parameters in the FFF 3D printing process impact the mechanical and fracture properties of continuous carbon fiber-reinforced thermoplastic composites (C-CFRTPCs). Focusing on raster patterns (±45º and 0º/90º) and build directions (XYZ, XZY, ZXY) using a Markforged Mark Two printer, the study assessed mode I fracture toughness and effective fracture energy. The study found that optimized raster patterns and build directions can minimize void growth and promote better layer fusion, which ultimately reduces the likelihood of inter-layer delamination. Results showed that the XYZ and ZXY build directions reduced void formation compared to XZY, while raster patterns also influenced void growth. Factors like fiber bridging and pull-out were linked to process parameters, impacting fracture behavior. Digital image correlation (DIC) and SEM inspections further analyzed crack-tip strain fields and fracture surfaces. These insights could help improve C-CFRTPC design to resist void-induced fractures. Lee K. et al. [7] explored the manufacturing defects of 3D-printed C-CFRTPC. They used rotational scanning-based micro-CT and laser ultrasonic testing (LUT) to evaluate the defects. Their work highlighted the importance of fiber orientation and fiber–matrix interaction in achieving mechanical consistency and minimizing defects. The study determined the optimal carbon fiber content and set a radius for cylindrical 3D printing. To create a cylinder-shaped composite structure, they used a Markforged X7 3D printer for continuous fiber reinforcement (carbon) and Onyx (short carbon fiber/Nylon) as the matrix. NDT is essential for examining products with inherent defects, and LUT is the primary technique for evaluating flaws in two cylindrical CFRTPC specimens that were 3D printed [73]. To identify flaws during the fabrication of cylindrical continuous fiber structures, the orientation was configured so that the cylinder’s axis was aligned perpendicular to the print bed. Controlling printing orientation and fiber distribution ensures lower void content, improved dimensional accuracy, and enhanced mechanical properties. The size, type, and severity of defects in 3D-printed FRTPC materials are closely linked to process parameters, including nozzle temperature, print speed, layer orientation, raster patterns, build direction, filament feed rate, and material composition. Table 1 outlines common defects such as warping, delamination, porosity, and surface roughness, along with methods to control them, summarizing their impact on mechanical performance, dimensional stability, and structural integrity.

**Table 1:** Summary of defects in 3D-printed FRTPC materials.

Material/Filler (Process)	Defect	Control Strategies	Main Outcomes	Ref.
PEEK/CF Composite (FFF)	Delamination	Use of low-viscosity resin, pre-impregnation, and in situ laser preheating	Lower viscosity matrix improved interlaminar shear strength (ILSS), with higher CF content further enhancing ILSS. Macro pores were noted in the microstructure. Optimal interlayer bonding was achieved using 10 W laser power and 120 mm/min scanning speed.	[74,75]
ABS/CF Composites (FFF)	Warpage	Mounting an additional heating element on the printer head	Auxiliary heating at 160 °C with a 0° raster angle effectively prevented warpage, yielding a 31% rise in tensile strength and a 439% increase in ductility. This heating also reduced strain, enhancing fatigue resistance and minimizing anisotropy.	[76]
PLA/Copper Fiber (FFF)	Surface Roughness	Laser polishing	After laser polishing with a 5 W laser and a 200 μm beam diameter, surface roughness decreased by over 91%. Significant improvements in storage modulus, loss modulus, and Tg were observed, due to improved interfacial adhesion between PLA and copper fibers.	[75,77]
PLA, ABS, PP, PETG (FFF)	Porosity	Optimizing extrusion parameters (multiplier, wall thickness, internal fill, and temperature)	Neither polymer type nor temperature significantly influenced porosity. However, they optimized extrusion settings and verified G-code-reduced porosity, improving part quality. Greater homogeneity in intermediate wall layers increased part impermeability.	[71]
PP/Glass Fiber Composites (FFF)	Warpage	Use of glass fiber-reinforced, nanophase-separated PP blends (Catalloy resins)	Post-annealing, Catalloy PP/GF composites exhibited minimal warpage (1.60%), compared to higher warpage in Copo-PP/GF (11.1%) and Homo-PP/GF (18.2%). These composites matched the ABS benchmark warpage (0.07%) without reducing heat-deflection temperature.	[75,78]
CFRTPCs (FFF)	Fiber Misalignment	Optimizing nozzle path and feed rate	Adjusting the nozzle path and reducing the feed rate helped reduce fiber misalignment, improving the tensile and load-bearing capacity of the printed components.	[33,41]
Nylon/CFRTPC (FFF)	Matrix Cracking	Increasing fiber content and optimizing layer orientation	Increasing fiber content from 30% to 50% and optimizing layer orientation minimized matrix cracking, leading to enhanced flexural and impact strength of the printed components.	[33,79,80]
Onyx/CFRTPC (FFF)	Incomplete Fiber Impregnation	Pre-heating fiber filaments and optimizing print speed	Pre-heating the fiber filaments and reducing the print speed improved fiber impregnation and eliminated voids, resulting in higher overall mechanical properties and reduced failure in tensile and bending tests.	[81,82]
CFRTPCs/Robot-Assisted Laser Additive Manufacturing (RLAM)	Porosity	Robot-assisted laser heating combined with roller compaction	The RLAM process achieved a porosity level of 0.19%, comparable to autoclave standards, with enhanced mechanical properties: flexural strength of 584 MPa.	[83]

**Figure 8:**
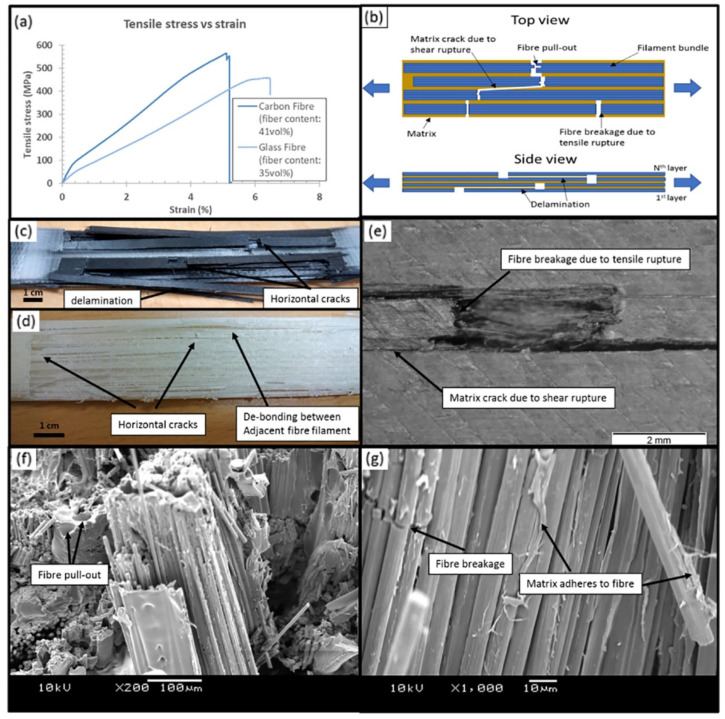
(**a**) Stress–strain curves for additively manufactured carbon and glass FRTPCs, (**b**) Illustration of tensile-fracture mechanism, (**c**) Fracture mode of carbon fiber-tensile specimen, (**d**) Fracture mode of glass fiber-tensile specimen, (**e**) Matrix crack due to shear rupture and fiber breakage from tensile rupture, (**f**) SEM image displaying fiber pull-out at the fracture surface, (**g**) SEM image showing fiber breakage with matrix adhesion to the fiber. Reproduced with permission from [84], © 2018 Elsevier.

Table 2 presents an overview of manufacturing defects and associated process parameters in FRTPCs. It systematically categorizes controlled parameters such as the filler/matrix ratio, highlighting identified characterizations of defects like voids, delamination, and fiber misalignment. Moreover, it delineates the limitations of existing research, providing a holistic understanding of the challenges and opportunities in FRTPC manufacturing. Goh et al. [84] examined the fracture behavior of 3D-printed carbon and glass fiber-reinforced composites through flexural, tensile, and indentation tests. Their study highlighted the potential of FFF for producing cost-effective C-FRTPCs with short manufacturing times, focusing on both microstructural features and mechanical performance. The chosen carbon and glass fibers represented distinct cost considerations due to their varying raw material costs. The study examined tensile, flexural, and indentation resistance for sports, automotive, and aerospace applications, with SEM and micro-CT scans revealing microstructural details and fracture mechanisms. The micrographs revealed voids within the extruded filament, likely due to inadequate impregnation or consolidation processes, impacting the composite’s porosity. Mechanical tests unveiled stress–strain behaviors and fracture mechanisms, showcasing the dominance of fiber-related failure modes and the efficiency of load transfer from matrix to fibers. SEM images, as depicted in Figure 8, highlighted extensive fiber breakage and matrix–fiber interfacial bonding, underscoring the composite’s mechanical integrity. Despite some challenges, like fiber pull-out attributed to fabrication processes, the study underscored the potential of additively manufactured C-FRTPCs for high-performance structural applications. While acknowledging the limitations, such as the slow deposition rate compared to conventional methods, the study emphasized the complementarity of additive manufacturing with existing composite fabrication processes. This comprehensive evaluation contributed valuable insights to product designers considering FFF techniques for continuous fiber composite part fabrication, highlighting both opportunities and challenges in realizing cost-effective and customizable composite solutions.

**Table 2:** Overview of manufacturing defects and process parameters in FRTPCs.

Filler/Matrix	Controlled Parameters	Identified Characterizations	Limitations of Research	Ref.
ABS	Raster pattern, width, direction, layer thickness, and air gap	The study focused on tensile, impact, and flexural strengths, but defect characterizations were not explicitly addressed.	Lack of detailed defect analysis may limit the understanding of the effect of process factors on defects.	[85]
PLA	Layer height and orientation	Emphasis on tensile and modulus, while defect analysis was not a primary focus.	Limited insight into how process parameters influence defect formation.	[86]
ABS	0°, 30°, 45°, 60° and 90° raster angles, and Horizontal, vertical, and perpendicular orientations	Surface roughness, tensile, and flexural strengths were analyzed, with orientation having a significant influence on defects, but defect types were not thoroughly explored.	The study primarily examined surface-related defects, potentially missing other types of flaws. The mechanical properties of the 3D-printed material are not sufficient for different applications such as UAS structures.	[87]
ABS	Neck growth between layers	Tensile strength was studied in relation to neck growth, while comprehensive defect characterizations were not conducted.	Limited focus on the effect of other process constraints on defect formation. The material performance of the 3D-printed materials is not sufficient for different applications, such as UAS structures.	[88]
ABS and PLA	Infill density	Mechanical properties (flexural, tensile, compressive) were investigated, while defect analysis was not the primary objective.	Detailed defect characterizations were not provided, limiting the understanding of defect formation.	[89,90]
Tri-material (PLA-PETG-ABS)	Printing speed, infill density, and layer thickness	The influence of FFF-processing parameters on tensile properties, including tensile strength and tensile strain, was examined as part of the printed composite responses. SEM and optical microscopy revealed the presence of defects such as micropores, voids, and micro/macro delamination, which were particularly prevalent under conditions of high layer thickness, high printing speed, and low infill density.	Detailed defect characterizations were not provided, limiting the understanding of defect formation. The mechanical performance of the tri-material composites is inadequate for various applications, including use in UAS structures.	[91,92,93,94]
(CKF, CCF, CGF)/Nylon	Fiber-volume content, layer thickness, and build direction	A wide range of mechanical tests were conducted, but specific defect characterizations were not a central aspect of the study.	Lack of explicit focus on defect characterization and identification.	
SCF/PLA	Various layer thicknesses and printing environment	Interlaminar properties were examined, with defect characterizations not explicitly discussed.	Limited information on how process parameters contribute to specific defect types.	[95]
CF/PLA	Printing parameters (temperature, speed, orientations, and layer thickness)	Creep behaviors were studied, alongside tensile, impact, friction, and wear properties, but defect identification was not the focus.	Defect characterization was not a primary objective, potentially leading to a limited understanding of defects’ influence on properties.	[96]

**Figure 9:**
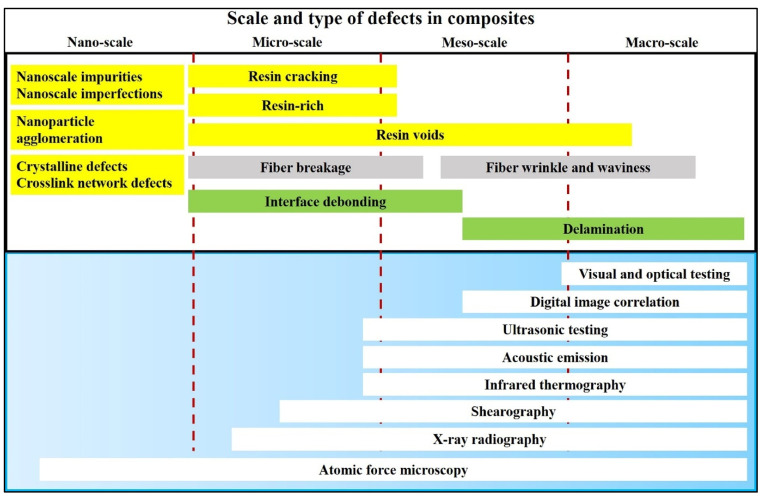
Types and scales of defects in composites alongside the corresponding detection methods [57]. Reproduced with permission under CC BY 4.0, © 2022 Elsevier.

The limitations of 3D-printed polymers and short fiber-reinforced polymers, such as their low mechanical performance, have been overcome by incorporating continuous fiber reinforcement into pure and short fiber-reinforced plastic composites. However, 3D-printed continuous FRTPCs still face fundamental challenges related to manufacturing defects, notably the presence of excessive microscopic and macroscopic voids. In a study conducted by Q. He et al. [46], optical microscopy (OM) and micro-CT were employed to quantitatively evaluate the detrimental effects of microscopic voids in 3D-printed continuous carbon fiber-reinforced nylon (CF/PA6) composites. The findings showed that these voids, both within and between filaments, significantly impair mechanical properties such as tensile strength, flexural performance, and interlaminar strength. This underscores the critical need for developing in-process strategies to minimize void formation in continuous fiber-reinforced composites during 3D printing, which is essential for broadening their application in various industries. Fu et al. [57] conducted an in-depth analysis of manufacturing defects and detection techniques in fiber-reinforced resin matrix composites, which are increasingly critical in the aerospace and automotive sectors. Due to the complexity of the materials and molding processes, these composites are prone to defects such as residual stresses, voids, and resin-rich areas. The study examined resin curing and infiltration methods, including hot pressing, resin-transfer molding, and 3D printing, to understand resin-rich defects. It also explored fiber defects like wrinkles, waviness, and misalignment, as well as their negative effects on structural properties. Interfacial issues such as delamination and debonding were analyzed, focusing on their causes and interface optimization. Additionally, machining defects, particularly those from drilling and cutting, were shown to result in issues like delamination, tearing, and burrs, all of which compromise the strength and reliability of component connections. The study also presented non-contact detection methods for identifying defects at different scales, aiding in the prediction of damage and lifespan in composites (Figure 9). Lastly, the analysis covered defects in various composite types, including laminated, woven, braided, and additively manufactured forms, concluding with future perspectives on improving composite manufacturing and defect-detection methods. Table 3 provides a concise summary of recent articles focusing on multi-scale manufacturing-induced defects, covering materials, processes, major findings, and limitations. Oztan C. et al. [97] investigated the microstructures of 3D-printed continuous fiber composites, including unidirectional carbon and Kevlar fibers in a nylon matrix, to correlate them with mechanical properties like stiffness and strength. Tensile properties were evaluated alongside a comparison to expected values, revealing approximately 30–40% weaker strength and stiffness in the printed composites compared to traditionally produced counterparts. This discrepancy was attributed to imperfections at the interfaces between printed layers, the presence of micro voids, incomplete fill density, and other process-induced artifacts. The study used thermogravimetric analysis to assess fiber-volume ratios and employed SEM and OM to examine and characterize the hierarchical microstructure. The analysis identified incomplete layer fusion, resin voids, gaps in the print structure, and fiber–matrix interface defects. It found weak bonding between fiber tows and the matrix, leading to poor mechanical properties. The study emphasized the need for process optimization and post-processing to improve composite performance and highlighted the importance of addressing microstructural defects to fully realize 3D printing’s potential in engineering applications.

**Table 3:** Evaluation of recently published articles on the materials, process, major outcomes, and their drawbacks in understanding multi-scale manufacturing-induced defects.

Materials	Processing	Research Outcomes	Drawbacks of the Research	Ref.
**Non-modified PVC filament**	Adjusting FDM parameters, such as nozzle diameter, layer thickness, print speed, and raster angle	Identified high-density cavities as the primary sources of crack propagation and failure in FDM parts. A broad range of mechanical properties was achieved, with tensile and flexural strengths most affected. Raster angle and printing speed had the greatest impact on results.	No in-depth analysis of multi-scale defects (<100 μm) resulting from FDM	[98]
**Continuous carbon fiber-reinforced polymer (C-CFRP) composites**	Novel 3D-printing extruder using in situ impregnation	Low void content achieved in C-CFRP composites. Excellent mechanical properties were observed due to proper carbon fiber impregnation. Thinner layers produced greater ironing forces, reducing voids and improving surface finish and mechanical characteristics.	High traction force led to reduced dimensional accuracy in areas with sharp curvatures. Lacked detailed multi-scale defect characterization.	[64]
**C-CF/PPS composites**	FDM with varying nozzle temperatures	High strength was achieved at both ends of the temperature range (310 °C and 330 °C), with the minimum strength at 320 °C. A correlation between strength, filament deformation, and void fraction was found. The strength was related to filament area fraction, showing filament deformation.	No clear trend between strength, crystallinity, and void fraction. No detailed multi-scale defect analysis.	[99]
**Short carbon fiber-reinforced polyamide (PA6) composites**	Three-dimensional printing (Fortus 900mc commercial printer) with different printing directions	Higher void volume in specimens with 0° printing directionDominance of connected voids in specimens with 0° printing direction Lower out-of-plane compressive resistance in 0° printed specimens compared to other printing directions	Lack of detailed analysis of the specific types and origins of voids. Limited discussion on the potential methods to mitigate void formation during printing.	[100]
**Continuous carbon fiber-reinforced PLA composites**	Modified FFF (desktop Reprap-Kossel 3D printer)	Continuous carbon fiber reinforcement improved the impact resistance of honeycomb structures. Experimental results aligned with finite-element simulations. Failure modes involved matrix–fiber interface failure and separation between adjacent print paths.	No thorough analysis of the mechanisms behind the failure modes. Limited discussion on improving the fiber–matrix interface strength.	[101]
**Continuous carbon fiber-reinforced epoxy and PETG composites**	FFF 3D printing process (Anisoprint composer A3 printer)	Achieved a maximum flexural strength of 294 MPa and modulus of 32.5 GPa. Micro-CT revealed void distribution and interfacial bonding. Dynamic mechanical analysis offered insights into thermo-mechanical properties. An optimal processing window was identified based on statistical analysis.	Defects and fiber-volume fraction limited mechanical performance. Further process optimization and nozzle design refinement are needed.	[102]
**Continuous carbon fiber-reinforced plastic**	FDM with path-planning algorithm	The proposed path-planning algorithm enabled continuous printing without filament cutting, enhancing efficiency. X-ray CT confirmed reduced gaps and improved bonding between adjacent vertices through parameter adjustments. The algorithm worked well for complex patterns and geometries.	Lacked a detailed analysis of multi-scale defects and their impact on mechanical properties. Challenges remained in optimizing algorithm parameters for further improving uniformity and strength.	[103]
**Continuous carbon fiber-reinforced polymer composites (CF/PA6)**	FDM 3D printing	High void content (up to 12%) was found in printed CF/PA6 composites, with poor fiber–matrix interfaces leading to reduced mechanical properties. Compression molding reduced void content to 6% and significantly enhanced mechanical strength. The study highlighted the potential of improving mechanical performance in continuous fiber composites.	Insufficient exploration of defect types in FDM processes for continuous fiber composites. Multi-scale defect characterization was limited, requiring further investigation to improve performance.	[46]
**Continuous carbon fiber-reinforced polyamide (C-CF/PA6) composites**	FDM (Prusa i3 MK3s)	High porosity and fiber misalignment resulted from weak interfaces and uneven pressure. Increasing angles and reducing curvature exacerbated defects, causing significant fiber breakage at turning angles above 120° or radii below 5 mm. Parametric studies showed that fiber bundle size and volume fraction influenced defect formation.	The current FE model could not directly detect certain defects like filament folding or fiber breakage. Further research is needed to fully capture and understand multi-scale defects in the printing process.	[41]

### 2.5. Classification of FFF-Induced Defects

**Figure 10:**
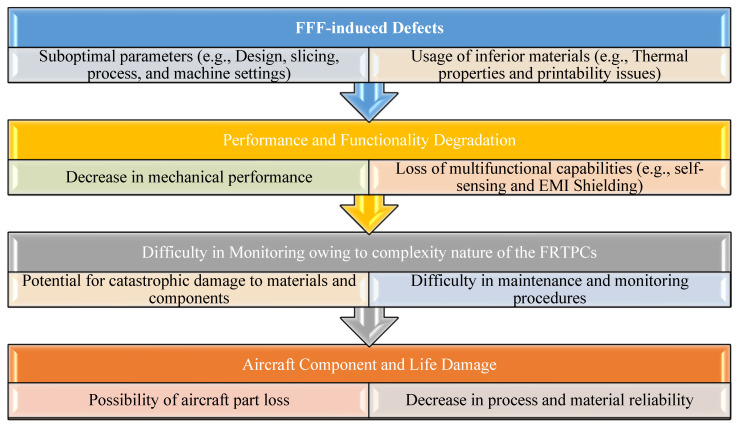
Impact of FFF manufacturing defects on FRTPCs across the manufacturing process to component failure [7,104].

**Figure 11:**
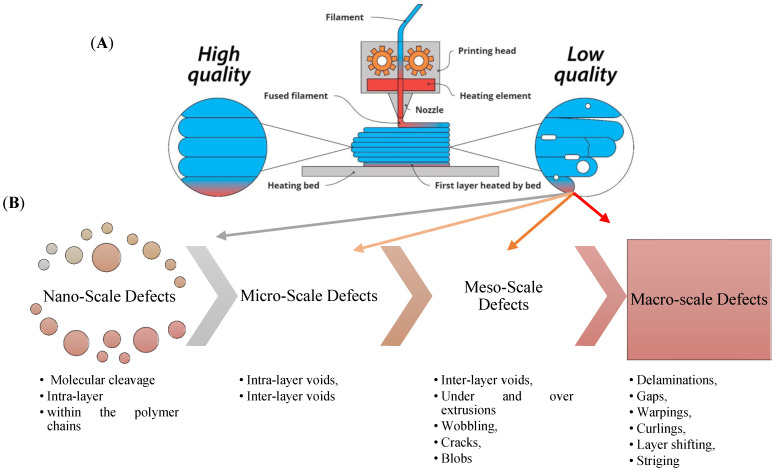
FFF processing (**A**), C-FRTPC defect classification and failure as a function of size scale (**B**) [104,105,106].

AM has become a transformative technology for producing complex structures using diverse materials, including FRTPCs. However, AM processes for FRTPCs are prone to defects that may undermine the mechanical properties and overall structural integrity of the fabricated parts. A thorough understanding of these defects is essential for improving quality control and optimizing the AM process. The key characteristics of defects in FRTPCs produced by AM include their morphology, location, and size [43]. Furthermore, FFF printing faces challenges related to suboptimal parameters, such as design flaws and improper slicing techniques, as well as the use of inferior materials with subpar thermal properties or poor printability [104,105]. These issues can result in defects like poor layer adhesion and nozzle clogging, compromising mechanical performance and multifunctional capabilities such as electromagnetic interference (EMI) shielding and self-sensing. Additionally, monitoring FFF-printed parts for defects presents difficulties, with the potential for catastrophic damage due to undetected defects like cracks or delamination. This is particularly concerning in industries like aerospace, where component failure could lead to aircraft part loss. Overcoming these challenges requires robust monitoring techniques and quality-control measures to ensure the reliability and safety of FFF-printed components throughout their lifecycle, while continuous improvement in printing parameters and material quality is essential for enhancing process and material reliability [7]. Figure 10 illustrates an overview of challenges encountered in FFF printing, encompassing suboptimal parameters, material issues, performance degradation, monitoring difficulties, and risks to components and lifecycle. Figure 11 depicts the FFF-printing process for high-quality (with no defect) and low-quality printing, as well as the generation of defects such as voids. In general, FFF-induced defects can be categorized based on (1) morphology or spatial topology, (2) location, (3) size, and (4) nature of occurrence (Table 4).

#### 2.5.1. Morphological/Spatial Topological-Based Classification of FFF-Induced Defects

Morphology refers to the shape, structure, and characteristics of defects formed during the additive manufacturing process of FRTPCs [106]. These defects can generally be classified into two categories based on their morphology: volumetric and planar. Volumetric Defects: Volumetric defects refer to irregularities within the material volume. These can include porosity and inclusions. Porosity occurs when voids or air pockets are trapped within the material during the deposition process. These voids can vary in shape, size, and distribution, affecting the overall density and mechanical behaviors of the FRTPC. Inclusions, on the other hand, are foreign particles or debris that become embedded in the material during the additive manufacturing process. These can arise from contamination of the feedstock material or from the environment in which the printing occurs. Planar Defects: Planar defects occur on surfaces or within layers of the printed part. They can include cracks and delamination. Cracks are discontinuities that extend through the material, compromising its structural integrity. Delamination describes the splitting of layers within FRTPCs, often resulting from weak bonding between layers or incomplete fusion during the additive manufacturing process.

#### 2.5.2. Location-Based Classification of FFF-Induced Defects

**Figure 12:**
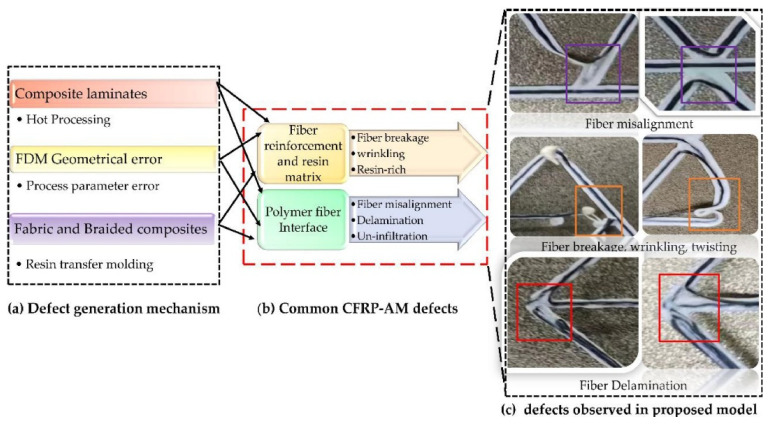
Surface defects observed in proposed C-CFRTPC–AM model. Reproduced with permission from [107], © 2024 Elsevier.

The position of defects within a printed part is crucial in assessing their severity and their effect on the structural integrity of the component [7]. Defects can be categorized by their location as follows: (1) Surface Defects: Surface defects occur on the outermost layers of the printed part and are open to the exterior environment. These defects can include surface roughness, pitting, and surface porosity (Figure 12). Surface defects are more readily visible and accessible for inspection but can still compromise the aesthetics and functionality of the part. (2) Subsurface Defects: Subsurface defects are located beneath the surface of the printed part but are still within the superficial layer. These defects are not immediately visible but can affect the mechanical behaviors of the parts. Examples include subsurface porosity and inclusions. (3) Internal Defects: Internal defects are located within the thickness of the material and are not visible from the exterior. These defects can include voids, inclusions, cracks, and delamination within the bulk of the FRTPC. Internal defects are particularly challenging to detect and characterize, but they can have substantial consequences for the structural integrity and performance of the printed component. Table 5 presents a comprehensive overview of characterization techniques, advantages, limitations/challenges, and future opportunities in defect-location analysis of FRTPCs in AM processes.

#### 2.5.3. Classification of FFF-Induced Defects Based on Their Size Range

The size of defects in AM of FRTPCs can vary significantly, ranging from a few nanometers to several millimeters [108]. The size of defects influences their severity and the extent of their impact on the mechanical behaviors of the printed component. (1) Microscale Defects: Microscale defects are characterized by dimensions on the order of nano-to-micrometers. These defects can include nanoscale voids, microcracks, and micro delamination. While individually small, microscale defects can collectively weaken the material and contribute to premature failure under mechanical loading. (2) Mesoscale Defects: Mesoscale defects are larger than microscale defects but smaller than macroscopic defects, typically ranging from tens to hundreds of micrometers in size. These defects can include larger voids, cracks, and delamination between layers. Mesoscale defects can notably weaken the mechanical properties of FRTPCs, resulting in decreased strength and stiffness. (3) Macroscale Defects: Macroscale defects are defects with dimensions on the order of millimeters or larger. These defects are readily noticeable without magnification and may consist of large cracks, voids, and delamination. Macroscale defects pose the most significant risk to the structural integrity of the printed part and often result in outright failure during mechanical testing or service conditions.

Clayton B. et al. [106] investigated failures and defects in FFF 3D-printed polymers and composites, identifying prevalent challenges such as warping, poor layer adhesion, surface imperfections, and material inconsistencies. The researchers proposed strategies for addressing these issues, focusing on the optimization of printing parameters and ensuring material quality control. Their findings provide valuable guidance for enhancing the reliability of FFF-manufactured components. Triyono J. et al. [109] examined how nozzle hole diameter affects porosity and tensile strength in PLA 3D-printed parts. Smaller nozzles reduced porosity and improved structural robustness, though an optimal size range was found for maximizing tensile strength. The study highlights the importance of selecting the right nozzle size and optimizing printing parameters for better quality and performance in PLA 3D printing.

## 3. Manufacturing Defect Detection and Characterization Techniques

Following the reconstruction efforts post-World War II, NDT emerged as an indispensable tool for ensuring the integrity and reliability of industrial materials and structures [43]. Currently, NDT plays a crucial role in the quality-assurance processes across various sectors, driving efficiency, safety, and innovation. NDT has since adapted to address evolving industrial materials and designs, including nanostructured materials and sophisticated composites. Advancements in computational modeling, signal processing, and sensor technology have enabled NDT methods to meet industry demands. NDT applications have expanded into biomedicine, renewable energy, and aviation, ensuring structural dependability, safety, and performance. NDT techniques mitigate risks and enhance inspection performance in various fields, underscoring their continual evolution and impact on industrial processes, environmental protection, and human safety [43].

As researchers and industrial practitioners advance materials science, sensor technology, and data analytics, NDT will continue to shape the technological environment [110]. NDT comprises a variety of inspection methods that assess an object’s physical integrity without damaging or affecting its operation [43]. These methods help understand an object’s features and behaviors by providing qualitative and quantitative insights regarding flaws, including density, size, location, and form. Traditional and cutting-edge NDT procedures range from basic to complex. Standard testing methods include Visual Inspection Testing (VT), Magnetic Particle Testing (MT), Radiographic Testing (RT), Liquid Penetrant Testing (PT), Electromagnetic Testing (ET), Thermal/Infrared Testing (IR), Ultrasonic Testing (UT), and Acoustic Emission Testing (AE). NDT is essential for structural systems and equipment reliability and safety, from early material investigation to post-production assessments and continuous maintenance tests. Material and component integrity can be assessed using these signal-processing complexity-based conventional or advanced methods [43]. Conventional NDT procedures are decades old and used throughout sectors. Techniques include VT, PT, MT, RT, and UT. Visual inspection is the simplest NDT method, checking components for surface flaws. Using PT and MT, ferromagnetic materials are checked for surface-breaking flaws like cracks and discontinuities. Radiographic testing uses X-rays or gamma rays to find holes, inclusions, and cracks in component structures. Ultrasonic testing uses high-frequency sound waves to discover interior defects or thickness. However, contemporary NDT methods use cutting-edge technology and hardware to improve sensitivity, resolution, and efficiency. When conventional methods fail, these approaches inspect complex geometries, advanced materials, and critical components. Advanced NDT procedures include eddy-current testing (ECT), phased array ultrasonic testing (PAUT), guided wave testing (GWT), digital radiography (DR), computed tomography (CT), and acoustic emission (AE). In PAUT, multiple ultrasonic elements controlled by computer algorithms make and direct ultrasonic beams to precisely find and describe flaws. ECT uses electromagnetic induction to find flaws on and below the surface of conductive materials. It can check for non-ferromagnetic materials and complex shapes that are hard to describe. GWT uses low-frequency ultrasonic waves to evaluate pipelines and structures from afar. Digital radiography and computed tomography provide detailed cross-sectional images of components for flaw detection and investigation. Acoustic emission testing identifies stress waves from material faults or damage, assessing structural integrity in real time.

### 3.1. NDTs for FFF-Manufacturing Defects of FRTPCs

**Figure 13:**
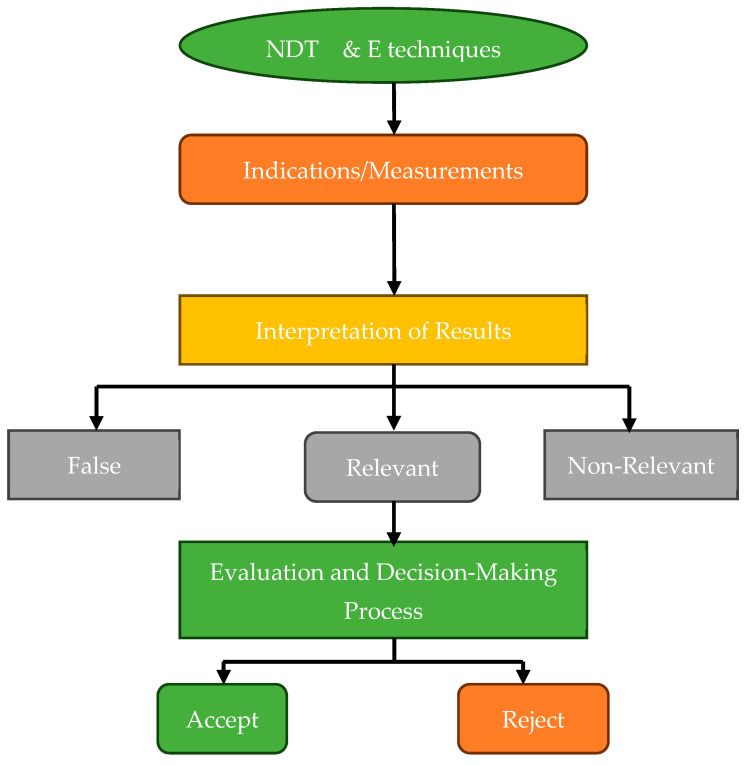
Illustration depicting the stages and decision-making procedure for applying NDT methods to evaluate the integrity of thick composite materials. Reproduced with permission from [117], © 2022 Springer Nature.

NDT techniques are extensively employed in the aerospace sector to assess and maintain the integrity of critical components, including UAS structures [111,112]. These methods are typically categorized as either conventional or advanced, with the classification largely based on the complexity of processing energy signals [43,113]. NDT serves as a valuable tool for inspecting raw materials prior to processing, assessing sub-components, scrutinizing finished products at various stages of production, and evaluating the integrity of structural systems and equipment during both operational and maintenance phases. Since the 1960s, NDT has undergone significant advancements, resulting in a transformational progression in the field of material inspection [114]. In this era, examining imperfections, including cracks, gaps, porousness, non-metallic inclusions, and forging laps, became a common activity made easier using NDT techniques [113]. In the last 50 years, this area has successfully adjusted to introducing new technical materials, implementing stricter quality standards, incorporating complex geometries, and increasing safety requirements [35,110,115]. Incorporating automation and computational tools has improved data gathering, storage, and processing skills in the NDT sector, contributing to its continuous development and significance [116]. NDT inspections use various chemical and physical energy to interact with the material. The approach relies on a variety of energy exchanges. NDT’s main ideas, inspection settings, chosen method, and a wide variety of characteristics show that finding material discontinuities is directly related to a sensitivity threshold. The inspection process’s sensitivity threshold strongly relates to the method’s core principles, inspection circumstances, and precisely calculated parameters. It standardizes NDT technology’s capacity to identify and analyze material defects. The complicated link between the sensitivity threshold and the fundamental aspect of NDT illustrates how accurate and dependable it is for discovering and characterizing material structural faults. Figure 13 illustrates the stages and decision-making procedure included in utilizing NDT techniques to assess the integrity of thick composite materials.

Data accuracy at different stages of the inspection method may be compromised due to intrinsic constraints [35,118]. These restrictions cover various topics, including the detector’s sensitivity, potential external interferences caused by signal generators, coupling effectiveness, and testing conditions like surface cleanliness. Signal amplification encounters limitations due to issues such as the instability of high-gain amplifiers, the need for frequent recalibration, and vulnerability to environmental variations, such as temperature swings. Difficulties intensify when addressing tiny imperfections, such as meso, micro, and nano defects, as it becomes challenging to differentiate changes in the probing medium caused by material contact due to interference from surrounding noise. Moreover, as certain features like resolution and picture quality are improved, the duration of measurements and operating expenses tend to increase. Proper calibration of the inspection determination to match the sample size and specific NDT parameters is crucial for accurate evaluation. Furthermore, there may be difficulties in reaching the area for investigation, mainly if it is located inside. Understanding fault characteristics is generally required for the effective implementation of NDT techniques. Notably, NDT results, whether quantitative or qualitative, should not be relied upon in isolation to determine the severity of a defect. This highlights the importance of data that provide insights into the consequences of flaws, the suitability of repair strategies, and other in-depth evaluations. Technologically, there is an ongoing scientific problem with conducting non-destructive assessments of tiny flaws (such as meso, micro, and nano defects) in various materials and components. Although there have been significant breakthroughs in materials engineering, microfabrication, and nanofabrication, the commercially available NDT methods have yet to keep up with these developments. This discrepancy underscores a need for more technical advancement within the NDT field, specifically examining minuscule flaws in contemporary materials and components [35,118].

Despite the increasing focus on utilizing FRTPCs in aerospace applications, particularly in UAS and aircraft structures, existing NDT techniques are still limited in their capability to detect internal damage or material degradation [39]. These techniques are often time-consuming, involve expensive equipment or high operating costs, require specialized training to operate or interpret the results, and can mostly detect surface-level defects at macro scales [63,112,119]. Duchene et al. [120] discussed the expanding importance of polymer composite materials in the critical construction safety and integrity of aerospace, railway, and wind turbine industries. Identifying the onset of subcritical damage is crucial for ensuring safety and reducing costs. NDT has become indispensable for monitoring (both in situ and ex situ) mechanical damage in composite materials. They analyzed the strengths and weaknesses of major NDT approaches, emphasizing that no single method is sufficient to diagnose all forms of mechanical damage [43]. Thus, the choice of NDT technology is determined by the nature of the damage processes and operational conditions. An interdisciplinary approach, utilizing a combination of NDT techniques, is recommended to achieve a more precise and comprehensive assessment of structural damage in polymer composite materials [43,121].

The primary NDT method in composites is ultrasonic testing, traditionally relying on a contact transducer and couplant for acoustic impedance matching, with limitations related to automation and the need for expert inspectors [7]. In contrast, LUT provides a fully non-contact method, making it particularly useful in difficult or high-temperature environments. LUT identifies defects by producing ultrasonic waves using pulsed lasers, which can propagate as either volumetric or surface waves. The frequency band of these waves depends on the laser pulse width. LUT’s advantage lies in its ability to provide highly detailed inspection results through an advanced automation system, without constraints on the target size or shape. Recently, Silva et al. [63] conducted an experimental and multicriteria comparison of four NDT methods: pulse thermography, ultrasound with air coupling, terahertz continuous wave, and digital radiography. The study aimed to characterize a 0.5 mm-thick artificially inserted defect in unidirectional continuous carbon, glass, and Kevlar fiber-reinforced PLA composite produced using conventional FFF 3D printing. Table 6 summarizes the limitations and defect-size-detection ranges for different NDT methods. This information helps professionals select the most appropriate method based on their specific inspection needs and constraints.

Detecting defects in CFRTPCs using NDT methods is vital for ensuring the reliability and safety of manufactured components. Various techniques have been explored to identify the smallest defects in AM components. For example, eddy-current testing can detect inner defects as small as 5 mm in diameter at a depth of 0.5 mm, achieving a signal-to-noise ratio of 2 or greater at a frequency of 250 kHz [122]. Resonant acoustics and phased array ultrasonic testing (PAUT) can detect vertical cylinder flaws up to 200 µm in size [123]. Computed tomography (CT) and digital X-rays are highly effective for inspecting intricate 3D geometries, capable of detecting defects potentially smaller than 10 µm in diameter, making them well-suited for aerospace applications [124]. Industrial CT testing improves accuracy by removing artifacts and using comparison test blocks [125]. Random-forest classifiers in XCT enhance the detection of micro porosity and cracks, identifying defects close to the voxel size [126]. In metal additive manufacturing, flying laser-scanning thermography detects flaws on rough surfaces, showing potential benefits and limitations [127]. Eddy-current probes are capable of detecting subsurface defects in stainless steel and titanium, with the smallest identified defect being an artificially created notch measuring 0.07 mm in width and 25 mm in length, along with blind holes ranging from 0.17 mm to 0.3 mm in radius (Table 6) [128]. NDT methods such as laser-ultrasonic, acoustic emission, optical emission spectroscopy, and thermography are employed in Wire and Arc (WAAM) and fusion welding (FW) to detect defects that affect material properties and may lead to component failure [129]. Integrating multiple NDT methods within a smart manufacturing process enhances defect detection and reduces testing time and costs, focusing on defective volumes in CFRP laminate samples [130]. The smallest detectable defect size in CFRTPCs using NDT methods ranges from 100 µm to 0.5 mm, depending on the specific technique and application context. Ultrasonic testing, when combined with CNN-based terahertz (THz)-signal processing, can detect micro defects smaller than 20 μm in GFRP composites [131]. Ultrasonic C-scan analysis effectively identifies defects within CFRPs and GFRPs [132]. Laser-line thermography identifies defect sizes and geometric positions in CFRP materials, controlling characterization error within 2.2% and achieving depth classification accuracy of 97% [133,134]. For inspecting polymer matrix composites (PMCs) with unidirectional fibers, digital X-ray, continuous-wave terahertz, air-coupled ultrasound, and active pulse thermography serve as benchmark techniques, detecting artificial delamination as thin as 0.5 mm [63]. Coefficient clustering analysis (CCA) in pulsed thermographic inspection is used to assess damage in CFRP laminates, offering improved visual confirmation and precise measurement of the damage size [135]. Millimeter-wave (mm-wave) imaging offers higher resolution and dynamic range in flaw detection compared to traditional Fourier methods [136]. Vision-based methods, thermography, and ultrasound inspection are compared for their resolution, sensitivity, and measuring thresholds, suggesting that integrating these techniques could optimize defect characterization in composite materials [137]. The diversity and complementarity of NDT techniques underscore the importance of selecting appropriate methods based on specific defect characteristics and material properties to ensure comprehensive evaluation and quality assurance of CFRTPCs (Table 6). Ongoing advancements in NDT technologies are steadily improving the accuracy and fidelity of defect detection in composite materials [138,139]. Table 6 depicts various NDT technologies for detecting manufacturing defects in 3D-printed CFRTPCs, including their penetration depths, advantages, limitations, and defect-detection ranges.

**Table 6:** NDT methods for detecting manufacturing defects in 3D-printed CFRTPCs, including their penetration depths, advantages, limitations, and range of defect-size detection.

NDT Method	Penetration Depth	Advantages	Limitations	Range of Defect-Size Detection	Ref.
Radiographic Testing	Up to 300 mm	High penetration depth, capable of detecting internal and surface defects	Safety concerns due to radiation exposure, high equipment, and operational costs	Defects from 0.01 mm to several centimeters (e.g., voids, inclusions, cracks).	[63,124,140]
Ultrasonic Testing	Up to 50 mm (high frequency); up to 100 mm (low frequency)	High resolution, suitable for internal defects in composites and metals	Dependent on material properties and surface conditions; requires skilled interpretation	Defects from 0.1 mm to several cm (e.g., delamination, cracks, fiber pull-out, fiber misalignment, voids).	[7,68,110,113,132]
Eddy-Current Testing	Up to 5 mm	Fast inspection, sensitive to both surface and near-surface flaws	Limited to conductive materials, shallow penetration, and complex signal interpretation	Surface and subsurface defects, such as cracks, corrosion, and delamination, can extend up to 0.5 mm in depth and 5 mm in length.	[122,128,140,141,142]
Thermography	Up to 4 mm	Contactless inspection, effective for near-surface defects and suitable for large areas	Limited penetration depth, affected by surface emissivity and temperature variations	Minor surface defects (ranging from 0.1 to 0.5 mm) and subsurface defects up to 4 mm, such as delamination and impact damage.	[133,134]
Acoustic Emission	0.5-5 mm.	Real-time monitoring of large areas; sensitive to dynamic defect activities	Requires dynamic loading, complex data analysis, and skilled interpretation	Defects range from sub-mm to up to 5 mm, including crack propagation and fiber breakage.	[123,143]
Magnetic Particle Testing	Up to 3 mm	Simple, cost-effective, and suitable for detecting surface defects in ferromagnetic materials	Limited to ferromagnetic materials and surface-condition sensitivity	Defects typically larger than 50 μm (e.g., cracks, seams, laps).	[144]
Liquid Penetrant Testing	Up to 2–3 mm	Simple, cost-effective, and capable of detecting fine-surface defects	Limited to surface defects, requires clean and smooth surfaces	Defects from 0.1 mm to several millimeters (e.g., cracks, porosity, pinholes).	[145]
Terahertz Imaging	Up to 25 mm	High-resolution, non-ionizing radiation, effective for non-metallic materials	Limited penetration depth and high equipment cost	Defects from 0.1 mm to several millimeters (e.g., delamination, voids, inclusions).	[63,131,140,146]
Radio Frequency Testing	Up to 30 mm	Effective for layered structures and delamination detection	Surface-condition sensitivity and limited penetration in thick materials	Defects from 0.1 mm to several centimeters (e.g., delamination, voids).	[140,146]
Shearography	Up to 2–3 mm	Low noise, minimal operator training, effective for surface defects and delamination	Difficult to detect subsurface defects, often requires complementary methods	Surface defects and features up to 2-3 mm deep (e.g., delamination, debonding, surface damage).	[142,146]
Computed Tomography	Up to 0.1 µm (nano-CT)	High-precision 3D imaging; detailed internal structure analysis	Sample size affects resolution; limited field of view; high cost	Surface, subsurface, and internal defects (e.g., cracks, delamination, microscopic failures, fiber misalignments, voids, interlayer bonding, matrix cracking).	[124,140]
Electrostatic Transducer UT	1–5 mm	Good resolution, portable, quick scanning	Contact-based; complex setup; ineffective for deep flaws	Surface and subsurface defects within 1-5 mm (e.g., cracks, delamination, wall thickness variations).	[140,147]
Piezoelectric Transducer UT	Up to 25 mm	Flexible, wide bandwidth, and effective for non-porous materials	Limited high-temperature applications; requires coupling medium	Surface and subsurface defects up to 25 mm (e.g., cracks, delamination, wall thickness variations).	[146,147,148]
Frequency-modulated Continuous Wave	From 1000 mm to 1 mm (microwave), 1 mm to 35 µm (THz)	Non-contact, effective in harsh environments; good for surface and subsurface analysis	Limited penetration depth; spatial resolution constrained by bandwidth	Surface and subsurface defects from 35 µm to 1 mm (THz) and 1 mm to 1000 mm (microwave) (e.g., delamination, inclusions, foreign materials).	[136,140,146,147]
Visual Inspection	Limited to surface defects	Simple and cost-effective; immediate results	Limited to surface defects, low accuracy for small defects	Surface defects are typically larger than 1 mm (e.g., cracks, corrosion, surface damage).	[145]

Existing advanced NDT techniques exhibit a wide range of capabilities in detecting micro- and meso-scale defects across various materials and component sizes [43]. For instance, X-ray computed tomography (XCT) can detect pores, voids, and cracks down to ~1 µm in composites, wood-based materials, and metals. Similarly, X-ray computed laminography (CL) is used for metals and polymers, with a detection order of ~10 µm. Techniques like Micro-Laser Line Triangulation (Micro-LLT) and Micro-Laser Spot Thermography (Micro-LST) can identify micro porosities and cracks in steel and polymers, with resolutions reaching approximately 100 µm. Thermal Tomography Imaging (TTI) can pinpoint hotspots in metals and plastics with a resolution of ~1 µm, while Scanning Thermal Microscopy (SThM) and Micro-Raman spectroscopy can assess thermal properties and stresses in GaN down to ~50 nm. Other notable methods include digital holography and electronic speckle pattern interferometry (ESPI) for detecting micro fibers and scratches in polymers and glass, with resolutions around ~10 µm. Table 7 provides a thorough outline of these existing NDT methods, detailing their capabilities in terms of material compatibility, types of detectable defects, and the smallest order of defect size they can identify.

**Figure 14:**
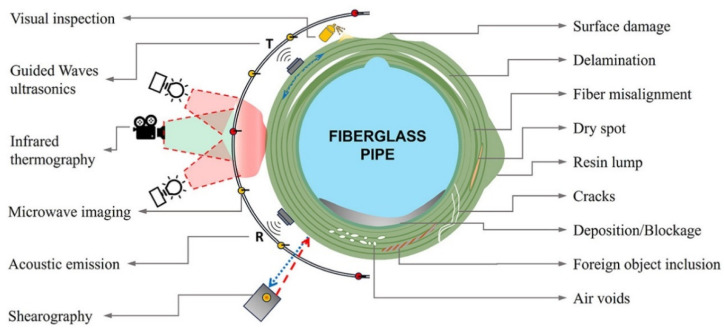
NDT techniques and their depth-specific capabilities for defect detection in fiber-reinforced polymer pipelines. Reproduced with permission from [149], © 2024 Elsevier.

Waqar M. et al. [149] conducted an in-depth analysis of NDT methods applied to fiber-reinforced polymer (FRP) pipelines, which are increasingly utilized in industries such as oil, gas, and water due to their superior corrosion resistance and favorable strength-to-weight ratio. The study emphasized the difficulties in detecting defects within FRP materials, as their non-homogeneous and anisotropic properties differ significantly from traditional metallic pipelines, reducing the effectiveness of conventional NDT techniques. The researchers examined a range of NDT approaches, including guided-wave ultrasonics, infrared thermography, and microwave imaging, evaluating their capability to detect subsurface defects at varying depths within the pipeline structure (see Figure 14). For example, guided-wave ultrasonics were useful for detecting deep-seated defects like delamination or fiber misalignment, while infrared thermography identified surface and near-surface anomalies such as resin lumps and dry spots. Microwave imaging showed potential in detecting internal defects, though it required further refinement for industrial applications. Despite progress in these methods, the review concluded that NDT for FRP pipelines was still developing. Significant research was needed to refine these techniques for more reliable early-defect detection and proactive monitoring. The emphasis was on shifting from reactive maintenance strategies, where issues were addressed post-failure, to proactive strategies that anticipated and prevented failures. This shift was vital for ensuring the safety and long-term reliability of FRP pipelines, particularly as their use continued to grow in critical industrial applications. The review called for a concerted effort in the research community to develop and validate NDT techniques tailored to the unique challenges posed by FRP materials. Figure 14 illustrates various NDT methods, such as guided-wave ultrasonics, infrared thermography, and microwave imaging, highlighting their effectiveness in detecting defects at different depths within FRP pipelines. The techniques were depicted in relation to their ability to identify subsurface anomalies like delamination, fiber misalignment, resin lumps, and dry spots, emphasizing the need for tailored NDT approaches for comprehensive pipeline monitoring.

**Figure 15:**
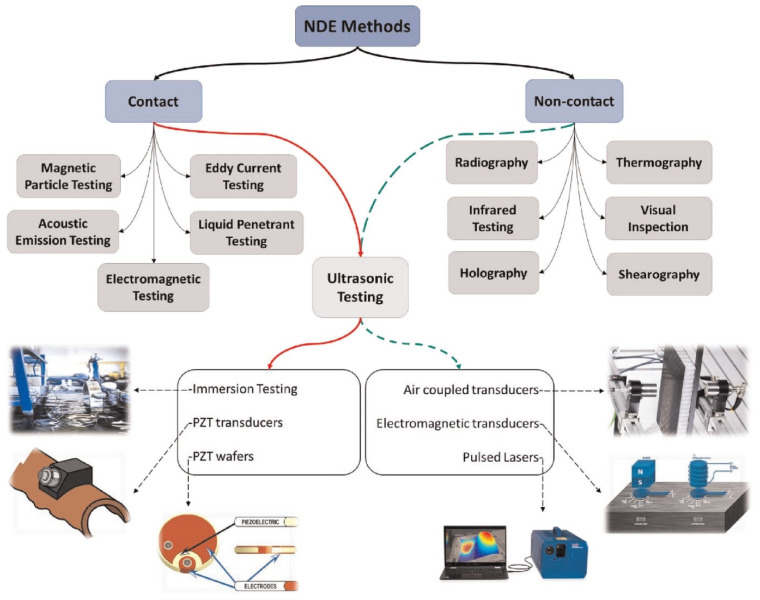
Diagram illustrating contact and non-contact, non-destructive testing methods. Reproduced with permission from [68], © 2024 SAGE Publications.

Mortada H. et al. [68] conducted a comprehensive review of non-contact ultrasonic-based non-destructive techniques for monitoring manufacturing defects in composite materials. Through an exhaustive examination of existing literature, the authors explored the effectiveness and applicability of various ultrasonic methods in detecting defects such as voids, delamination, and fiber misalignments. Their review critically evaluated the advantages and limitations of non-contact, non-destructive ultrasonic techniques compared to traditional contact-based methods, emphasizing their potential to enhance defect-detection capabilities and improve quality-assurance processes in composite manufacturing. Additionally, key challenges such as signal attenuation and limited penetration depth were identified, providing valuable insights into proposed strategies for overcoming these obstacles. The review emphasized the need for ongoing research and development to improve these techniques and overcome current limitations, thereby advancing non-destructive testing in composite materials. In contrast, contact methods like ECT, PT inspection, and MT require direct contact with the material surface for effective evaluation. Non-contact methods, like thermography and laser-based techniques, enable remote sensing without surface contact. These methods offer diverse advantages, including high sensitivity, rapid inspection, and suitability for different material types and inspection scenarios [68]. Figure 15 illustrates both contact and non-contact, non-destructive evaluation methods.

**Figure 16:**
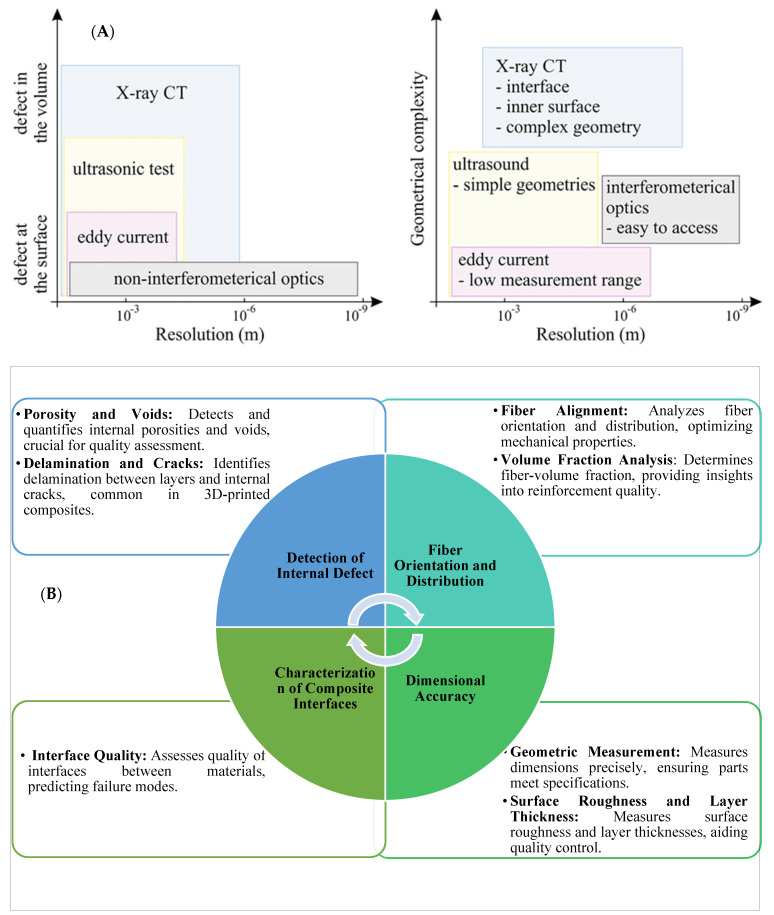
(**A**) Systematic classification of NDT techniques by defect location (**left**) and geometric complexity (**right**), (**B**) Utilization of X-ray CT for identifying defects in AM of CFRPCs [150]. Adapted with permission under CC BY 4.0, © 2020 Springer Nature.

Figure 16A presents a systematic classification of NDT techniques, organized by defect location (left) and geometric complexity (right). The left axis discerns the proficiency of these techniques in identifying defects at diverse locations within a material or structure, encompassing surface, subsurface, and volumetric dimensions. Concurrently, the right axis factors in the geometrical intricacy of structures, elucidating the adaptability of NDTs to varying complexities. This dual classification provides a scientific framework, aiding in the precise selection of NDTs tailored to specific defect characteristics and structural geometries [150]. Figure 16B illustrates the primary applications of X-ray CT in detecting and analyzing common manufacturing defects in the AM process, including porosity, delamination, fiber misalignment, and voids.

**Figure 17:**
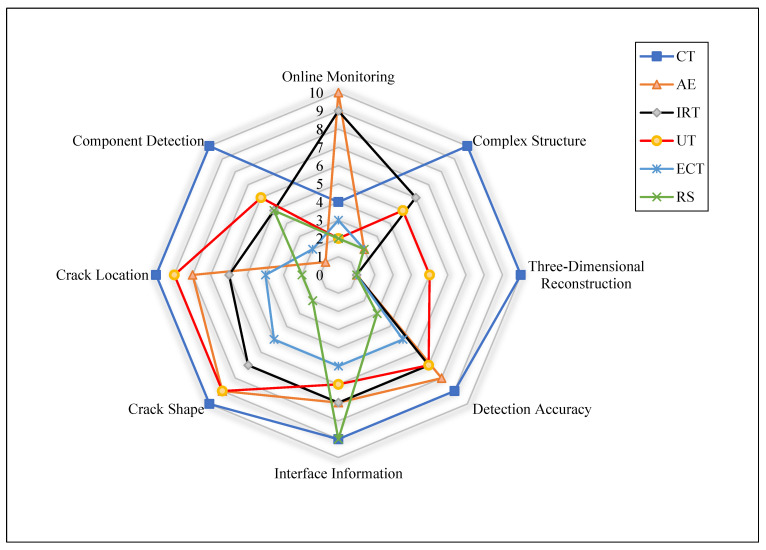
Comparison of NDT techniques for CFRTPCs, illustrating the efficacy of micro-CT and other NDT in defect detection and characterization. Reproduced with permission from [151], © 2024 Taylor and Francis.

Han S. et al. [151] conducted a review of NDT techniques applicable to CFRTPCs, emphasizing their significance in detecting inherent defects and ensuring material integrity. The study employed a multi-dimensional evaluation framework, comprising meta-analysis, case study metrics, and empirical data analysis, to compare the efficacy of various NDT methods, such as micro-CT, AE, UT, IRT, ECT, and RS, as depicted in Figure 17. Notably, micro-CT emerged as a standout technique, demonstrating superior performance in defect detection and characterization. The review also explored emerging trends in SHM technologies, underscoring their potential for real-time monitoring and predictive maintenance strategies. Challenges in SHM, including sensor integration and scalability issues, were discussed alongside future advancements driven by innovative sensor designs and Internet of Things (IoT) integration.

### 3.2. Self-Sensing for SHM

**Figure 18:**
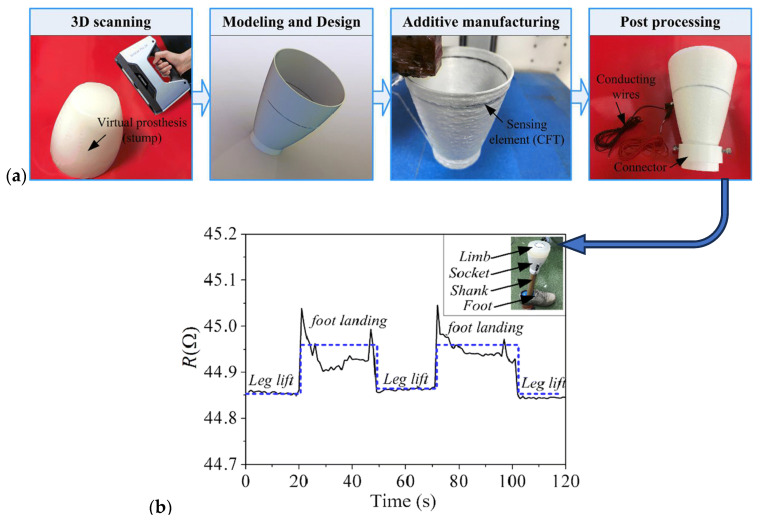
(**a**) Manufacturing process of the customized prosthetic plug and (**b**) electrical resistance variation across different states over time: leg lift and foot landing. Reproduced with permission from [158], © 2024 Springer Nature.

Conventional NDT techniques for UAS structures, such as visual inspection, ultrasonic testing, etc., have limited ability to control the fiber alignment and volume fraction, resulting in low consistency and suboptimal performance of the FRTPCs. To overcome these limitations, researchers have explored solutions through embedding and attaching fiber optic sensors such as fiber Bragg-grating sensors within the material during the fabrication process [5,152,153]. The fiber can detect changes in strain and temperature within the structure [154]. This promises real-time monitoring of the composite’s structural health, providing early warning of any potential damage or progress in defect size, which may otherwise lead to catastrophic failures during the in-service applications [155]. Nonetheless, embedded and attached sensors have disadvantages in terms of added weight and potential damage to the composite structure [156]. Embedded sensors are intrusive and may compromise the mechanical properties of FRTPCs. Similarly, attached sensors risk detachment, rendering them unsuitable for long-term use. In addition, they may adversely influence the performance of the FRTPC material in the UAS structures [156]. Additionally, the integration of embedded sensors is limited by the processing methods used for sensor embedding, mainly due to their extreme sensitivity to temperature and the potential for structural damage during the embedding process [157]. Luan C. et al. [158] developed an AM method for producing hybrid continuous carbon/glass fiber-reinforced thermoplastic composites with self-sensing capabilities. Mechanical and electrical tests showed that adding continuous carbon fibers enabled in situ SHM without weakening the composite’s strength. However, the integration of advanced SHM capabilities, particularly self-sensing technologies, significantly increases preparation and detection costs. The cost drivers include specialized AM equipment, precise fiber alignment, sensor calibration, and material compatibility testing. Despite the cost discrepancy, the long-term benefits of self-sensing systems—such as reduced maintenance, minimized downtime, and enhanced reliability—can make them economically viable for critical applications. The study highlighted a consistent change in electrical resistance within the elastic range and a notable shift upon structural damage, confirming the potential of carbon fibers as sensory elements in GFRTPCs. While the initial setup costs for self-sensing composites are high, they reduce the need for traditional NDT inspections and associated labor costs, making them an attractive long-term investment. The research suggested potential applications in the customization of prosthetic sockets [158]. Figure 18a describes the fabrication process of customized prosthetic sockets, detailing steps from 3D scanning for limb dimensions to 3D modeling and design with CAD software (version 23.1), employing a multi-material integrated AM method and concluding with post-processing steps integrating electrodes and conducting wires. Complementing this, Figure 18b visually represented the dynamic changes in electrical resistance during motion phases, showing a surge upon foot-ground contact, which was maintained until leg lift, and promptly reverting to a baseline post-leg lift, indicating a response to different states of motion.

However, the integration of advanced AM techniques with hybrid continuous carbon/glass fibers held potential beyond prosthetic socket customization. It contributed to the advancement of smart materials and their integration into composite structures, enhancing functionality and adaptability, with significant implications for material science and AM [155]. Conversely, Zhao Q. et al. [159] provided an extensive review of the electrical behavior of CFRTPCs, widely used in the aviation and civil industries. They highlighted the critical role of electrical conductivity in assessing the mechanical integrity and multifunctional applications of CFRTPCs, such as their susceptibility to lightning strikes and their potential for self-sensing. While recent research has primarily focused on the electrical conductivity of individual carbon fibers, the anisotropic nature of carbon fibers poses challenges in accurately measuring the electrical conductivity of CFRTPCs through bulk assessments. Therefore, the authors called for more accurate simulation models and microstructural studies to improve understanding of CFRTPCs’ conductive behavior and promote breakthroughs in CFRTPC development. This review offered valuable insights into the ongoing research on the electrical properties of CFRTPCs, underscoring the promising future of the CFRTPC industry. Gonçalves et al. [160] developed PEEK nanocomposite filaments infused with carbon nanotubes (CNT) and graphite nanoplates (GnP), designed for 3D printing using FDM technology. The filaments showed electrical conductivity between 1.5 and 13.1 S/m, with enhanced mechanical properties and greater thermal conductivity than PEEK. The addition of GnP improved melt processability, maintained desired electrical conductivity, and reduced the coefficient of friction by up to 60%. Although materials like CNTs and GnP offer significant performance advantages, their high cost remains a barrier to widespread adoption in SHM systems. The 3D-printed specimens had similar Young’s modulus and tensile strength to the filaments but exhibited lower strain at break and reduced electrical conductivity. The study suggests further optimization of 3D printing parameters to reduce porosity and improve electrical conductivity. A thorough analysis of carbon materials for cutting-edge uses in structural self-sensing, EMI shielding, and thermal interfaces across a range of sectors, including building, communication, lighting, and electronics, has been presented in [161]. The study emphasizes the creation of various high-performance, cost-effective carbon materials, such as carbon nanofiber, carbon black, exfoliated graphite, and carbon fiber. Flexible graphite and nickel-coated carbon nanofiber are ideal for EM shielding, while short carbon nanofiber cement–matrix composites and continuous carbon black polymer–matrix composites are especially effective for structural self-sensing applications. Furthermore, flexible graphite combined with exfoliated graphite paste, graphite nanoplatelet paste, and carbon fiber paste proves valuable for thermal interface applications. The review discusses the connected phenomena’s mechanics, as well as the standards for developing materials for these applications. Another recent review article by D.D.L. Chung [162] investigated self-sensing in carbon fiber-reinforced composite materials, where the material detected strain or damage without external sensors, reducing costs and improving durability. Carbon fibers provide electrical conductivity, enabling self-sensing through changes in conductivity. In polymer–matrix composites, longitudinal resistivity decreased under tension, while through-thickness resistivity increased. Flexural loading affected surface resistivity, and strain effects were reversible. However, fiber fractures or delamination caused irreversible increases in resistance. While self-sensing in CFRTPCs showed promising benefits, further research was needed to optimize performance and reduce costs associated with incorporating carbon nanofibers or nanotubes [162,163].

Despite the cost-related challenges, integrating advanced materials and SHM systems into CFRTPCs offers essential benefits, particularly in safety-critical applications like aerospace. In situ SHM ensures operational safety by providing early warnings of damage or deterioration through real-time monitoring. Traditional SHM methods, such as strain gauges, accelerometers, piezoelectric sensors, and fiber optics, measure parameters like strain and vibration but often suffer from high costs, reduced durability, and potential mechanical property loss [158]. Integrating sensors directly into composites creates multifunctional materials, preserving structural integrity while enabling continuous monitoring [164]. Continuous carbon fibers are especially promising, serving as both reinforcement and sensing elements by detecting changes in electrical resistance to monitor strain, stress, or fatigue. However, embedding such fibers into complex FRPC structures remains a significant challenge. Demonstrating the long-term savings and efficiency of these multifunctional composites through quantitative life-cycle analysis will be critical to promoting their adoption across industries.

Hwang M. et al. [165] demonstrated the fabrication of a piezoelectric glass GFRTPC as a smart material for impact sensing in SHM applications. While the study presents promising results, it also highlights certain limitations of the layup process in producing composites with high self-sensing capabilities. To address these limitations, it is recommended to use AM techniques like FDM for producing composites with aligned conductive fibers [166,167]. This can enhance the piezoresistive response of the composite, thereby improving its sensing capabilities for SHM applications. Furthermore, incorporating conductive fibers with high aspect ratios in the composite matrix can lead to improved electrical conductivity and piezoresistive response. This can enable the fabrication of high-performance FRTPCs with enhanced sensing capabilities for real-time SHM applications. The study conducted by Park et al. [112] focused on using in situ health-monitoring systems to evaluate damage in composite materials during tensile testing. The authors introduced a combined system that integrated infrared thermography with electrical resistance measurements. Additionally, a multiphysics simulation framework was developed to model the interaction of physical phenomena during three stages of damage: crack propagation, temperature changes, and electrical resistance variations. This electro-thermal-monitoring approach enabled the estimation of “damage stress” and the assessment of damage progression in GFRTPCs under quasi-static tensile loading. The research underscored the importance of understanding multiple physical processes during crack initiation and propagation. The main techniques used were infrared thermography and electrical resistance measurements, with GFRTPCs as the test material assessed through tensile testing to diagnose damage states.

**Figure 19:**
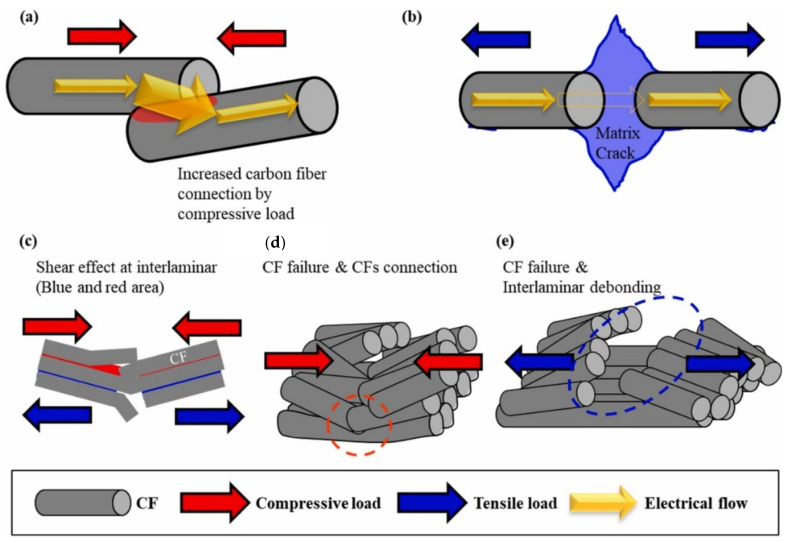
Illustrates a schematic diagram depicting the fractured shape of a double CF composite and unidirectional CFRTPC under various locations subjected to flexural load. Reproduced with permission from [169], © 2023 Elsevier.

Microscopic exploration of Change Electrical Resistance (CER) analysis is one of the crucial techniques for optimizing self-sensing outcomes of FRTPCs [162,168]. Shin et al. [169] employed CER analysis to investigate self-sensing capabilities in CFRTPCs. They conducted a comparative analysis between CER trends in CFRTPCs three-point bending and dual-fiber composite (DFC). The CER trends of DCF inserted at different positions were meticulously measured in the DFC specimen. The initial CER of the upper-side embedded carbon fiber (CF) experienced a reduction under compressive stress, ultimately leading to an increase in CER due to fracture. Figure 19 displays a schematic of fractured shapes in double-carbon fiber composites and unidirectional CFRTPC (UD-CFRTPC) under varied flexural load locations. During the application of tensile load from below, CF CER demonstrated enhancement over time. While the CER behavior of CFRTPC was comparable to that of DFC, the interface between two fragmented CFs modified it more significantly than in the case of DFC. DFC predicted the CER trend of CFRTPC on a microscale level, and the interface between CFs in composite materials greatly impacted CFRTPC’s ability to sense itself. At the micro scale, external forces elicited reactions in the CER, showcasing different trends under tensile and compressive forces compared to fracture behaviors. Comparisons between UD-CFRTPC CER and micro-scale results revealed that macro-scale resolution was 3–4.5 times lower than the micro scale. This study highlighted that UD-CFRTPC CER followed micro-scale trends, and the size of the CF interface altered the CER trend. The findings suggested that self-sensing in composite materials using electrical resistance (ER) should be enhanced based on the insights gained from this comprehensive analysis.

**Figure 20:**
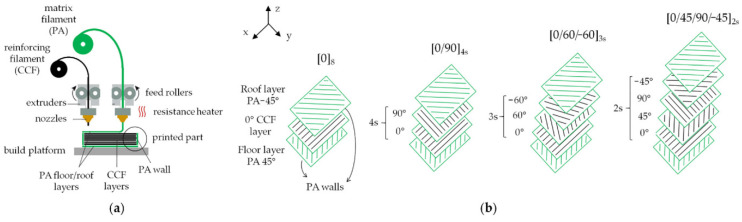
(**a**) Diagram illustrating the MarkForged^®^ FFF printing process; (**b**) internal structure of C-CF/PA composites, featuring PA roof and top layers, along with intermediate layers reinforced with C-CF. The fiber infill is oriented at varying angles (0°, 90°, 45°, 60°), based on the chosen layup configuration, with a PA contour surrounding each layer [51]. Reproduced with permission under CC BY 4.0, © 2022 MDPI.

Tabatabaeian A. et al. [170] provided a comprehensive analysis of mechanochromic self-reporting methodologies, focusing on their design principles and diverse applications. This study highlighted the potential of mechanochromic composites for structural health-monitoring systems. These composites, including color-changing materials, enhanced polymers, chromatic structural materials, and advanced hybrid-sensing systems, are distinguished by their capacity to visually signal structural integrity and detect damage in real time. This capability, combined with wireless functionality, sets them apart from traditional post-operation-monitoring methods, offering a significant advancement in SHM. The primary focus was on mechanochromic polymeric composites that reveal damage through optically induced changes caused by mechanical stress. Similarly, Wang et al. [51] investigated the mechanical and microstructural characteristics of 3D-printed C-CF/PA composites produced through the FFF process. Their results showed that the mechanical performance of these composites is largely dependent on the layup configuration and the microstructure formed during the FFF process. Specifically, composites with unidirectional fiber alignment in the loading direction exhibited higher stiffness and strength, while quasi-isotropic laminates predominantly displayed bead debonding. The study proposed that integrating microstructural and mechanical analysis with design optimization, supported by prognostic models and advanced production technologies, could significantly enhance the application of these composites in industries such as automotive and aerospace, where there is a need for lightweight, complex components with high load-bearing capabilities. Additionally, Shah et al. [171] conducted an extensive investigation of damage patterns in materials, analyzing signals and approaches for automated diagnostics. Their goal was to establish a taxonomy categorizing various damage types, including delamination, fiber breakage, and matrix cracking. The authors highlighted the importance of utilizing advanced manufacturing technologies and data-analysis techniques to improve the quality of 3D-printed composites. They further suggested employing ML and deep-learning algorithms for real-time detection of manufacturing defects, which could lead to significant enhancements in the reliability and performance of these materials. Figure 20 illustrates the MarkForged FFF process and the internal structure of the C-CF/PA studied by Wang et al. [51].

Non-destructive evaluation (NDE) methods, commonly used for post-damage analysis, are often time-consuming, providing insights into material degradation and structural integrity throughout the structure’s lifespan. Highlighted as a powerful alternative to conventional NDE techniques, SHM’s efficiency and reduced time requirements are emphasized [170]. It entails continuously observing systems over time, collecting response measurements from sensors either surface-mounted or integrated into composites during manufacturing, and offering novel perspectives on material deterioration and structural durability [163]. Successful NDT implementation, particularly in interior locations, necessitates prior knowledge of fault features and unrestricted access to the inspection area [43]. However, the qualitative or quantitative information obtained from NDT procedures often proves insufficient for evaluating defect severity, requiring a comprehensive understanding of defect consequences, appropriate repair methods, and additional crucial measures. Despite advancements in materials engineering, commercially available NDT solutions have lagged, posing a significant technical challenge. The primary objective is to unveil effective methods and new NDT approaches, fostering innovation to enhance accuracy and dependability in identifying tiny flaws, especially those less than 100 µm in size. This exploration encompasses assessing defect characteristics and analyzing penetration depth, permitted material types for inspection, spatial resolution, and the expected time of test procedures.

In the realm of material innovation, mechanochromic composites with self-reporting capabilities, as outlined in Table 8, have emerged as versatile assets for SHM [170]. These state-of-the-art materials offer instantaneous feedback on the structural integrity of various engineering structures, spanning concrete, steel, asphalt, and composites [43,172]. Their advantages over conventional SHM techniques are two-fold: they monitor systems throughout operations, avoiding post-operation delays, and operate wirelessly, eliminating the need for cumbersome data-acquisition systems [139,172]. The potential applications of self-reporting materials extend beyond monitoring, offering opportunities for integrated design concepts [173]. For instance, FRTPCs can serve dual functions as both structural elements and for SHM, eliminating the need for additional sensors. Glass fibers, incorporating fluorescent proteins and embedded in epoxy, can also serve as load-bearing elements and indicators for impact-induced delamination [174]. The utilization of self-reporting materials ensures effective monitoring and revolutionizes traditional sensor designs, heralding a new era of materials-led innovation in monitoring structural integrity [175].

**Figure 21:**
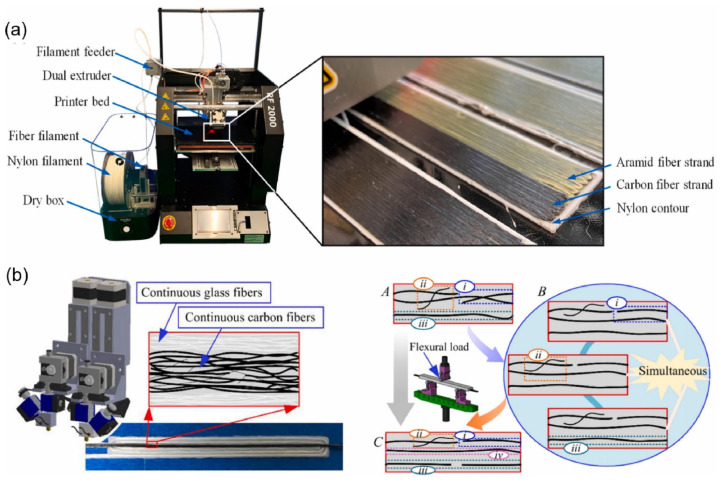
(**a**,**b**) Diagram and picture of hybrid continuous composites during the 3D printing process utilizing intra-layer hybrid methods. Reproduced with permission from [155], © 2024 Elsevier.

The development of self-sensing fibers (intrinsically smart) is an area of active research, and there are many potential avenues for exploring their properties. As the field evolves, it is liable that self-sensing fibers will become an increasingly important component of FRTPC materials and SHM systems [156,162,178]. The incorporation of self-sensing fibers in composites offers several advantages compared to attached or embedded sensors. Self-sensing fibers are less prone to damage or failure, as, unlike external sensors, they are less likely to break or detach from the structure. Additionally, self-sensing fibers can be integrated more readily into composite materials without requiring extra steps or equipment for sensor attachment [178]. Furthermore, self-sensing fibers offer a promising approach for the development of SHM systems in composite materials, particularly in FRTPCs, as they provide simple and reliable means of detecting changes in mechanical loads and temperature within the material [179]. Figure 21 shows hybrid continuous composites throughout the printing process using intra-layer hybrid methods, as illustrated in the schematic representation (a) and supported by an accompanying image (b).

In addition to the physical properties of fibers, the way they are arranged in the composite can also affect their self-sensing abilities. For example, fibers that are aligned in a certain direction can be more sensitive to changes in strain along that direction. By strategically designing the fiber orientation in the composite, the material can be made more sensitive to specific types of strain or deformation [180]. Furthermore, it is worth noting that conventional methods for manufacturing CFRTPC often lack the requisite control and adaptability needed to construct specific geometries, which are essential for incorporating unique inlays or smart provisions in structural features. In contrast, AM is readily posed to enable such features due to its significant level of flexibility and controllability [181]. AM offers a superior degree of control in the fabrication process, enabling the integration of conductive fibers and strategic fiber orientation to augment the associated self-sensing properties. Such precise control and adaptability are challenging to achieve through conventional manufacturing methods. Consequently, AM emerges as a promising approach for developing composites with advanced self-sensing functionalities [163]. The use of self-sensing fibers in FRTPCs can greatly improve their SHM capabilities without the need for additional attached or embedded sensors. This can lead to cost savings, reduced weight, and increased durability of composite materials. Fibers can also potentially demonstrate self-sensing properties through their piezoelectric or pyroelectric characteristics [182]. Piezoelectric materials produce an electric charge when exposed to mechanical stress, whereas pyroelectric materials produce an electric charge in reaction to temperature fluctuations [182,183]. Some types of fibers, such as quartz or polyvinylidene fluoride (PVDF), exhibit these properties and can be used to create self-sensing composites [184]. The addition of conductive fibers to thermoplastic composites can have a considerable influence on the performance of wing structures in UAS. However, a key consideration when selecting such conductive fibers is their inherent compatibility with the existing reinforcing fibers used in CFRTPC or the common choices of matrix (polymeric) materials. Conductive fibers that are not compatible with the matrix material or reinforcing fibers can cause delamination or degrade the mechanical behavior of the composites. Conductive fibers embedded in composite materials provide EMI shielding and electrical conductivity, making these composites ideal for applications requiring such properties, including communication and sensing systems [183]. Moreover, conductive FRTPCs can improve the structural integrity of wing components by enhancing both stiffness and strength [185,186]. The use of conductive fibers in wing structures can also improve the overall durability and longevity of UASs by reducing the risk of damage caused by lightning strikes and static discharge [178].

CNTs have been incorporated into FRTPCs to create conductive pathways that can be used to sense temperature and strain. Similarly, carbon fibers have been used as strain sensors in FRTPCs due to their piezoresistive properties [182]. Graphene has also been studied as a potential sensor material due to its high electrical conductivity and sensitivity to strain [187]. Reduced graphene oxide (rGO) and MXenes are other examples of conductive fibers that can function as both reinforcement and sensors in FRTPCs [188]. MXenes phases, derived from etching A layers in MAX phases, are innovative 2D materials, expanding the field of 2D materials with significant potential [189,190]. In addition to improving mechanical properties, they also provide the ability to sense and respond to mechanical loads [187]. Thus, the choice of conductive fibers must be made with careful consideration to ensure compatibility with both the matrix material and reinforcing fibers. This selection can significantly improve the mechanical and sensing properties of the composite material in UAS applications. FRTPCs can be produced via AM, such as FFF, leading to faster production times, reduced waste, and greater design flexibility [186]. These FRTPCs can incorporate conductive fibers as both reinforcement and sensors, allowing for real-time monitoring of structural health and damage detection. This synergetic approach can enhance the overall performance of the UAS structure by allowing for early detection and mitigation of potential structural failures, significantly improving reliability and safety. Additionally, these composites are lightweight and can be tailored to specific applications, making them an efficient solution and cost-effective for UAS structures. Overall, the use of self-monitoring additively manufactured FRTPC with conductive fibers has the potential to revolutionize the UAS industry by enabling the development of more advanced and reliable UAS structures [191]. Table 9 and Table 10 present a comprehensive analysis of piezoresistive-sensing approaches and defect-detection strategies in FRTPCs, offering valuable insights for researchers and practitioners in the field. Table 9 provides a comparative analysis of four different piezoresistive-sensing approaches, based on the chosen strategy [186], highlighting differences in sensitivity, manufacturing complexity, and limitations. Notably, the self-sensing method in carbon-FRTPCs offers ease of fabrication but limited sensitivity customization, primarily detecting fiber-dominated failure modes. Contrarily, piezoresistive matrices exhibit tailorable sensitivity within defined limits, making them versatile for specific applications but potentially overlooking fiber-dominated failures. Additionally, Table 10 delves into various defect-detection methods, underscoring the tailored sensitivity, manufacturing convenience, and performance spectra of carbon fiber composites with self-sensing, surface-deposited/mounted sensors, embedded filaments/yarns, and tailorable piezoresistive matrices.

Composites with self-monitoring capabilities have been developed by leveraging the electromechanical properties of continuous carbon fibers, which act as both reinforcement and sensors by detecting conductivity changes under mechanical load. Ye et al. [168] demonstrated that a carbon fiber-reinforced honeycomb structure could identify strain and damage through variations in electrical resistance during cyclic compression. Similarly, Luan et al. [194] designed a lattice truss structure that monitored strain and stress and predicted damage using carbon fibers. These results suggest that continuously reinforced composites offer strong self-monitoring potential for aerospace and automotive applications [33]. Table 11 summarizes the pros and cons of the key techniques used for void characterization. It systematically presents each characterization technique alongside its measurable characteristics, highlighting the respective pros and cons.

**Figure 22:**
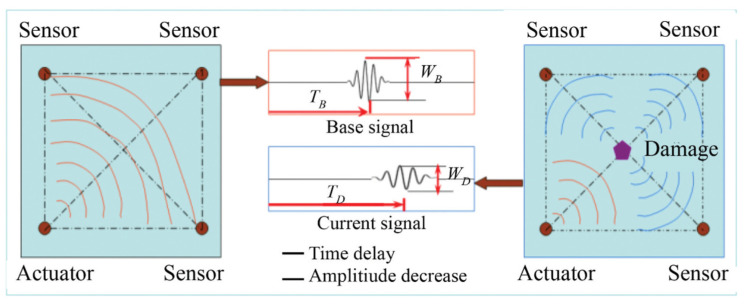
Schematic representation of damage-monitoring technology utilizing ultrasonic-guided waves [121]. Reproduced with permission under CC BY 4.0, © 2022 Taylor & Francis.

Researchers have explored combining SHM systems with NDT methods to measure the condition and integrity of AM-produced components [195]. The intricate geometries and tiny defects created by advanced AM techniques present challenges for traditional NDT approaches. Although CT is considered highly effective for identifying defects, its use is limited by its high expense and potential radiation hazards. LUT offers a practical alternative, providing high-resolution inspection like CT but at a lower cost and capable of handling complex geometries. LUT uses laser pulses to produce ultrasonic waves in the material, causing localized heating and expansion, which produces the waves [195]. These ultrasonic signals travel through the material, interacting with internal defects before being detected on the surface by a continuous wave laser (Figure 22). LUT’s key advantages include non-contact operation for faster inspections, no reliance on coupling agents, broadband detection for more comprehensive information, and high spatial resolution in a compact system.

### 3.3. 2D and 3D Imaging for Defect Detection in FFF-Fabricated FRTPCs

The microstructure of FRTPCs has a profound impact on their mechanical properties, making it a key factor in material design and engineering [118]. Traditional 2D imaging methods fall short of delivering complete spatial data about the structure under examination. In contrast, 3D imaging techniques offer a more comprehensive analysis, providing in-depth insights into the material. Three-dimensional imaging enables the evaluation of volume, connectivity of inhomogeneities, shape, and spatial size distribution, offering a more holistic perspective. Table 12 provides a thorough comparison of 2D and 3D imaging in aerospace applications. The analysis encompasses spatial information, defect identification, structural complexity, manufacturing optimization, material composition, engineering decision-making, in situ applications, and the influence on research and development.

The shift from 2D to 3D imaging is crucial, empowering researchers with a deeper understanding of material microstructures [43,196,197]. In a scenario where 2D radiographs confirm structural irregularities, subsequent 3D imaging not only confirms their existence but also enables precise localization and identification of their origin (Table 13) [197]. This multidimensional insight is instrumental in refining material designs, optimizing engineering processes, and ensuring product integrity. Moreover, 3D imaging provides actionable insights for material optimization, revealing the spatial arrangement and connectivity of inhomogeneities. This knowledge is invaluable for tailoring materials to meet specific performance criteria, enhancing mechanical strength, durability, and overall functionality. Identifying and localizing flaws through 3D imaging empowers engineers to strategically address issues, facilitating targeted modifications in material composition, manufacturing processes, or structural designs [198]. This proactive approach, driven by comprehensive 3D insights, contributes to the advancement of more robust and reliable composites. In the context of ongoing advancements in material science, the integration of state-of-the-art 3D imaging technologies accelerates innovation. Researchers can explore and understand the intricate nuances of material microstructures, pushing the boundaries of material design. This iterative process of exploration, identification, and refinement, facilitated by 3D imaging, fosters continuous improvement in the field of material engineering [197]. The shift from 2D to 3D imaging not only overcomes the constraints of conventional techniques but also paves the way for significant breakthroughs in material science and engineering. The ability to explore and comprehend the three-dimensional intricacies of material microstructures empowers researchers and engineers to make informed decisions, driving innovation and ensuring the development of materials with superior performance and reliability [199,200].

**Figure 23:**
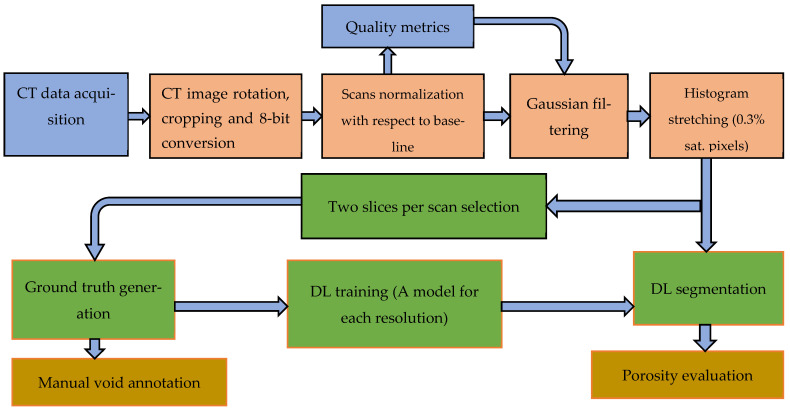
Workflow for image segmentation, including data acquisition (blue), image pre-processing (red), segmentation (green), and porosity evaluation (grey) [201]. Reproduced with permission under CC BY 4.0, © 2023 SAGE Publications.

Hernandez et al. [201] examined the effect of XCT scan settings on the evaluation of porosity in carbon fiber-reinforced plastic laminates. The study assessed various scan durations (ranging from 30 s to 60 min) and voxel sizes (6 to 50 µm), analyzing their impact on porosity measurements within a unidirectional carbon fiber epoxy composite. They found that as voxel size increased, smaller voids became harder to detect, and porosity was overestimated when resolution exceeded 25 µm due to partial volume effects. Scan times under 2 min produced noisy images that required aggressive filtering, impacting void-segmentation accuracy. Both thresholding and deep-learning techniques were employed for porosity segmentation, with deep learning proving more effective in recognizing noise and delivering more reliable outcomes. The research identified optimal scan parameters (voxel size ≤ 25 µm, scan times of 2 to 8 min) and deep learning as the most suitable segmentation method for capturing micro voids within the aerospace tolerance of 2%. Additionally, shorter scan times were found to improve CT scanning efficiency. Figure 23 outlines the image-segmentation process, detailing steps from data acquisition to porosity evaluation [201], providing a clear approach for accurate porosity assessment in carbon fiber-reinforced plastic laminates.

### 3.4. Micro Computed Tomography (Micro-CT)

**Figure 24:**
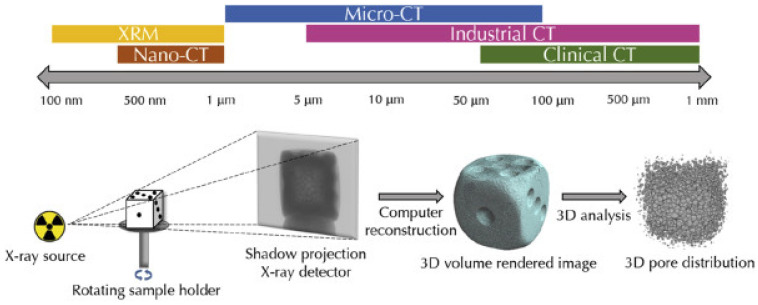
Overview of the operating principles of X-ray tomography, illustrating the characteristic length scales of current CT methods [118]. Reproduced with permission under CC BY 4.0, © 2020 Elsevier.

Micro CT has emerged as a cutting-edge X-ray imaging technique, recognized for its high resolution. It employs a cone-beam geometry with an X-ray tube and a rotating sample holder [118]. Unlike common CT used in life sciences or low-resolution, high-energy CT techniques common in industrial applications, micro-CT has gained substantial popularity in materials science. This growing interest is due to its versatility and non-destructive nature, enabling both in situ and operando analyses. Micro CT is also instrumental in the development and validation of computational models for materials. In a review by Vasarhelyi et al. [118], micro-CT was introduced, highlighting recent advancements and suggesting potential future applications. The review covered essential technical elements such as magnification, resolution, and the Hounsfield unit, providing a full understanding of the technology. In the context of AM, micro-CT is particularly valuable for identifying defects and characterizing continuous FRTPCs. The precision advanced by micro-CT in defect identification aligns with the demands of AM processes, where thorough characterization is crucial for optimizing manufacturing and ensuring structural integrity (Figure 24). Continuous FRTPCs, which are crucial materials in aerospace applications, derive significant benefits from micro-CT’s capabilities. This technique allows for a thorough examination of the spatial distribution and connectivity of fibers within the composite, offering valuable insights for optimizing material performance. This is paramount for tailoring materials to meet stringent performance criteria, thereby enhancing mechanical strength, durability, and overall functionality in the demanding environments of aircraft and unmanned aerial systems (UAS). Micro CT underscores its dual role as a valuable tool in materials science and as a crucial element in characterizing AM defects, particularly in the context of continuous FRTPCs. The detailed insights it provides into material microstructures and defects position micro-CT as a catalyst for innovation and optimization in both imaging technology and materials science [202].

**Figure 25:**
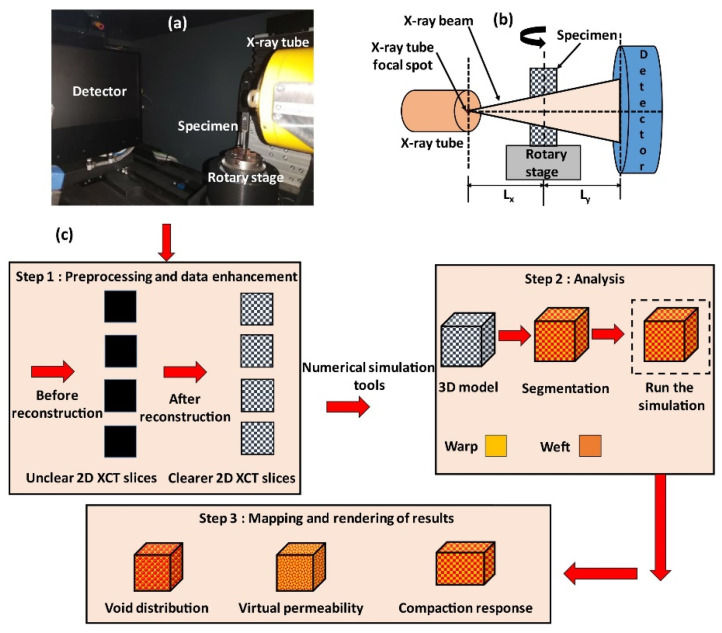
(**a**) Laboratory-scale XCT setup, (**b**) schematic illustration of the setup, and (**c**) step-by-step procedure for XCT data analysis [23]. Reproduced with permission under CC BY 4.0, © 2020 Elsevier.

**Figure 26:**
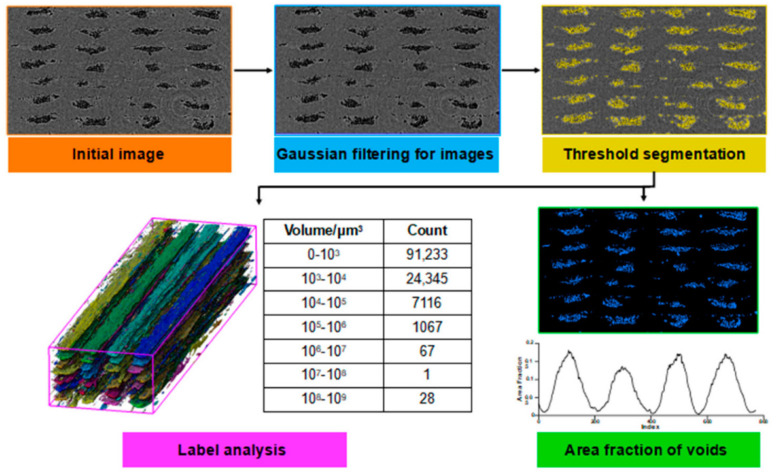
Steps of void extraction and quantitative analysis [65]. Reproduced with permission under CC BY 4.0, © 2023 MDPI.

Naresh et al. [23] extensively explored XCT applications in aerospace composite manufacturing, examining recent advancements, principles, and limitations. The review delved into challenges associated with predicting composite manufacturing parameters, particularly in Autoclave and Out-of-Autoclave processes, using XCT-derived material models. The imperative of optimizing aerospace composite manufacturing parameters was underscored, with XCT’s commendable spatial resolution and swift acquisition capabilities facilitating in situ monitoring for generating numerical models. Various modeling approaches, spanning meso scale to macro scale, were employed with XCT to predict diverse process parameters. In a laboratory setting, XCT functioned as a non-destructive evaluation tool, where the process of generating 2D radiographs and employing reconstruction algorithms, such as filtered back projection, was explained. Key parameters affecting accuracy and computation time during image processing were considered [23]. The integration of XCT with Computational Fluid Dynamics (CFD) in aerospace manufacturing techniques was advocated for its potential to craft advanced material models, ushering in a new era of improved aerospace manufacturing processes. For further clarification, Figure 25 depicts a comprehensive overview of the XCT setup, its schematic representation, and the stepwise XCT data-analysis procedure [23]. Wu et al. [65] conducted an in-depth investigation of the complex interplay among processing parameters and manufacturing defects in 3D-printed thermoplastic composites. Using advanced micro-CT technology, they performed detailed micro-scale analyses on CFRTPC (of PLA matrix) specimens, shedding light on key aspects of defect formation. One notable finding was the uneven distribution of fibers within the printed filament. Adjusting the filament feed rate from 100% to 50% led to an a/b ratio closer to 3.33, signaling better fiber alignment. However, this enhancement was offset by a sharp rise in porosity, which increased from 7.077% to 25.352%. Furthermore, analyses at a layer thickness of 0.2 mm revealed a delicate trade-off: while greater nozzle pressure decreased porosity, it also increased the likelihood of damaging fiber bundles. These findings, supported by quantitative evidence, shed light on the complex interplay between processing parameters and defect manifestation, offering valuable insights for refining manufacturing processes and mitigating defects in 3D-printed composite structures. Figure 26 delineates the sequential procedures for void extraction and quantitative analysis, encapsulating the essence of the study’s rigorous methodology.

**Figure 27:**
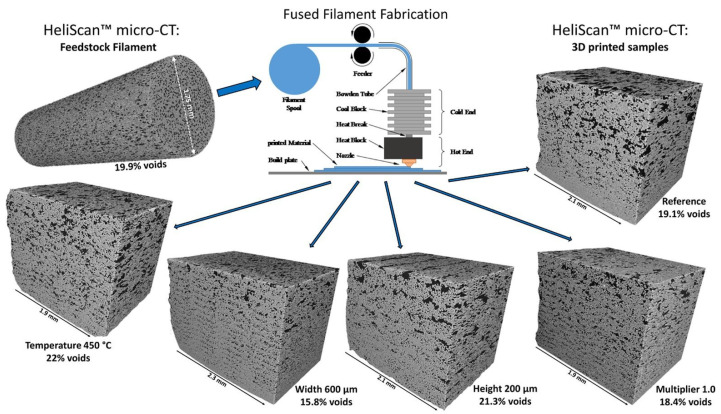
Application of XCT in the void detection and characterizations of FFF 3D-printed FRTPCs under various parameters. Reproduced with permission from [203], © 2024 Elsevier.

S. Sommacal et al. [203] examined the internal structure of 3D-printed CF/PEEK composites using XCT to assess void and fiber distribution. The study found that the feedstock filament contained a significant void volume (~19.9 vol%) with heterogeneous distribution. Analysis showed that the printing process did not eliminate these voids, as void content remained high (15.8% to 22%) across all five printed samples, regardless of printing parameters. The layer-by-layer printing process notably affected the alignment of voids and fibers, orienting them parallel to the mold plate. The findings provide valuable insights into the challenges and opportunities for optimizing the manufacturing process of carbon fiber/PEEK composites, setting the stage for future research in this domain. Additionally, Figure 27 demonstrates the application of XCT in the detection and characterization of voids in FFF 3D printed FRTPCs under various parameters. This visual representation highlights micro-CT imaging’s ability to offer detailed insights into void distribution and characteristics within printed composite materials. The application of XCT in this context underscores its value as a powerful non-destructive analysis tool, enhancing understanding of how various printing parameters influence void formation in FRTPCs [203].

### 3.5. Defect Control Strategies for FFF-Printed CFRTPCs

The fabrication of CFRTPCs using FFF introduces several defects that significantly impact the mechanical performance, dimensional accuracy, and durability of the printed parts. These defects include inter-layer and intra-layer voids, fiber pullout and breakage, matrix cracking, porosity, delamination, and surface imperfections. Controlling these defects is essential to maintaining structural integrity, minimizing anisotropy, and ensuring the reliability of components, particularly in critical applications like aerospace and automotive industries. Table 14 summarizes key defect types and advanced control mechanisms. Pre-deposition strategies, such as optimizing toolpaths and bead widths, reduce inter-layer voids by ensuring consistent material flow and layer fusion [11]. In situ techniques, including ultrasonic vibration and laser-assisted bonding, improve layer adhesion and minimize intra-layer voids caused by trapped air or irregular cooling. Fiber pullout and breakage are mitigated through surface treatments that enhance fiber–matrix adhesion and optimized printing paths that prevent misalignment and shear failure. Post-processing methods, such as thermal annealing and surface ironing, improve matrix bonding, relieve residual stresses, and enhance surface finish. Real-time feedback systems and monitoring technologies, including micro-CT imaging and acoustic emission testing, can offer precise defect detection and quality control. The comprehensive use of pre-deposition, in situ, and post-processing strategies ensures effective defect management, resulting in improved mechanical properties, dimensional stability, and the long-term reliability of CFRTPC components. Additionally, AI-based monitoring, ultrasonic-assisted printing, and post-process annealing play critical roles in producing high-quality parts [107]. Real-time feedback systems and structural health-monitoring (SHM) techniques provide continuous defect detection during service, enhancing the reliability and longevity of components. This multi-faceted approach addresses both micro- and macro-scale defects, significantly improving the performance and durability of CFRTPC parts.

### 3.6. Modeling Defects in FFF-Fabricated FRTPCs

Both traditional manufacturing and AM techniques tend to reduce the impact performance of FRTPCs, largely due to variations in fabric reinforcements, base materials, and process conditions. These inconsistencies often lead to manufacturing defects such as voids, delamination, fiber misalignment, and poor resin impregnation, all of which negatively impact the overall performance of the composite [54,204]. Defects like voids within FRTPCs can significantly degrade their impact resistance, limiting their versatility in various applications [163]. Additionally, there remains a lack of research in finite-element modeling (FEM) that accounts for the effect of voids on FRTPC impact performance [205]. In a recent review by et al. [204] on the use of ML in composite manufacturing, it was noted that 80% of ML models relied solely on synthetic data for training and validation. In contrast, only 12% of studies used an experimental approach, and just 8% combined both synthetic and experimental data. SHM techniques, such as NDT-based approaches, ML-based algorithms, and Design of Experiments methodologies, work in tandem to provide a comprehensive framework for evaluating and optimizing the integrity of various engineering structures. NDT-based SHM techniques such as micro-CT integrating with self-reporting FRTPCs, as the cornerstone, facilitate non-invasive assessment, allowing for real-time monitoring of structural health. ML-based algorithms contribute to advanced data analysis, leveraging patterns for predictive insights and early detection of potential issues. The integration of Design of Experiments methodologies, such as full factorial design, which can allow us to study the interaction of two and three parameters at the same time, ensures a systematic and efficient approach to experimentation, optimizing processes and variables. This trihybrid approach, encompassing diverse SHM techniques, delivers a robust strategy for effective monitoring, defect detection, and performance enhancement across industries [204]. This effort holds the potential to reveal the significant impact of various manufacturing defects on the performance of FRTPCs, providing crucial insights for future research and development.

#### 3.6.1. ML Algorithms for Assessing FFF-Manufacturing Defects in FRTPCs

**Figure 28:**
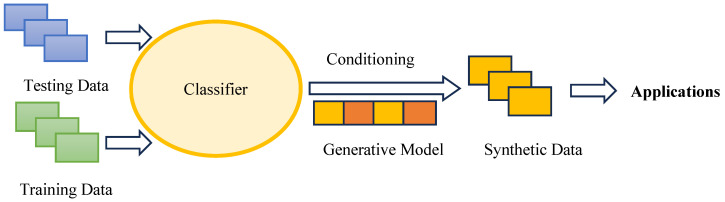
Synthetic data generation [209].

Machine learning (ML) is a specialized field within artificial intelligence (AI) that involves creating algorithms and models that enable machines to learn from data and improve their performance on tasks without being explicitly programmed. ML relies on algorithms and data to allow systems to autonomously learn and enhance their performance over time through experience. A key subfield of ML, deep learning, leverages intricate neural networks with multiple layers to achieve remarkable accuracy in complex tasks such as object recognition, speech processing, and language translation. Notable deep-learning models include feedforward neural networks, convolutional neural networks, autoencoders, deep-belief networks, and recurrent neural networks, all of which contribute to advancements in fields requiring high levels of precision and adaptability. [206,207]. ML comprises a suite of algorithms and methodologies employed in crafting systems that can acquire knowledge from data. These systems have the capability to make predictions or infer patterns from the available data [208]. The random-forest (RF) algorithm is an ensemble learning method that leverages the technique of bootstrap aggregating, or bagging, where multiple decision trees are used as base estimators. This approach enhances model accuracy by reducing variance and preventing overfitting. It combines the outputs of multiple decision trees, each typically a weak learner, to form a more accurate and robust model. RF stands out as a versatile ML algorithm that can be applied effectively in both classification and regression tasks [195]. Its increasing popularity is attributed to its robust performance, ability to prevent overfitting, and scalability. Furthermore, it can outperform deep neural networks in applications involving structured data with well-defined features or when the quantity of available data is limited. Figure 28 demonstrates the method employed to create artificial data for the purpose of training and testing ML models [209]. Synthetic data generation is a valuable technique in data science, allowing researchers to augment existing datasets or simulate scenarios that may be challenging to capture real-world data.

While FFF offers advantages in fabricating complex CFRTPC components, predicting their mechanical properties remains challenging due to various design factors. Conventional methods like finite-element analysis (FEA) struggle to accurately model the mechanical behavior, particularly for C-CFRTPCs. To overcome this challenge, the study by Zhang Z. et al. [210] aimed to predict the flexural strength of additively manufactured C-CFRTPCs using an ensemble learning-based predictive modeling method. This method combined multiple ML algorithms to enhance prediction accuracy compared to physics-based approaches. Experimental data from four-point flexural tests were utilized to validate the predictive model. The study demonstrated that an ensemble learning approach, combining various algorithms such as linear regression methods (e.g., least absolute shrinkage and selection operator (lasso)), spline-based techniques (like multivariate adaptive regression splines (MARS) and generalized additive models (GAM) with smoothing splines), instance-based methods (including support vector machines (SVM) and K-nearest neighbors (KNN)), and Tree-based models (such as extremely randomized trees (Extra Trees) and extreme gradient boosting (XGBoost, version, v3)), yielded highly accurate predictions of flexural strength. The model achieved a minimum root-mean-square error (RMSE) of 9.87 and a maximum coefficient of determination (R^2^) of 96.99%. Additionally, the research explored how design parameters, including the number of fiber layers, concentric fiber rings, and polymer infill patterns, impacted flexural strength. The findings indicated that increasing both the number of concentric rings and fiber layers significantly improved flexural strength. For example, flexural strength rose from 81.53 MPa to 269.11 MPa as the number of fiber layers increased from 2 to 18. This research highlights the potential of ML in predicting mechanical properties and optimizing design parameters for additively manufactured C-CFRTPCs. Future studies could explore additional design and AM parameters to further enhance predictive accuracy and understanding of structure–property relationships in C-CFRTPCs.

#### 3.6.2. ML-Enabled NDT Technique for FFF-Manufacturing-Defect Characterization

**Figure 29:**
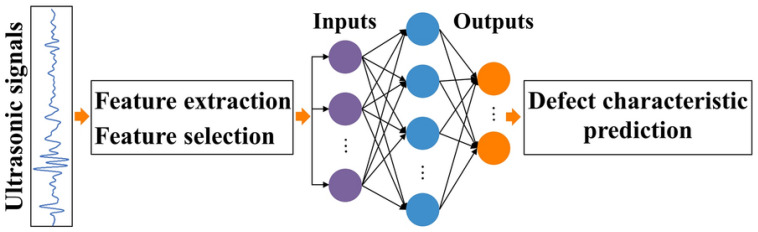
Overview of ML’s role in UT, including signal processing, feature selection, and defect prediction [211,213].

Shi et al. [211] investigated the use of an explainable ensemble tree model for detecting pipeline leakage via vibration signals, emphasizing the importance of interpretability in ML for NDT, particularly UT. They employed Shapley additive explanation (SHAP) to clarify the contributions of various features to the leakage-detection results, addressing the “black-box” issue of traditional ML models. This approach improved the understanding and reliability of the model’s predictions, crucial for practical applications. The study proposed utilizing advanced signal-processing techniques to extract multi-domain features from ultrasonic testing (UT) signals, alongside a feature-selection method based on model interpretation (FS-MIS). This method combined SHAP values with filter, embedded, and wrapper techniques to optimize feature selection. This method enhanced both accuracy and computational efficiency in defect detection. Experiments validated the framework’s effectiveness, showing significant improvements in detection accuracy and resolution. The research underscored the potential of interpretable ML models to advance UT technologies by optimizing feature selection and enhancing model transparency. A related study by Ye J. et al. [212] explored the advancements in applying AI to UT data interpretation for non-destructive evaluation (NDE). The researchers focused on overcoming significant challenges in the field, particularly the scarcity of publicly available annotated datasets and the absence of standardized performance benchmarks for deep learning models. To address this, they introduced the “USimgAIST” dataset, which comprises over 7,000 annotated ultrasonic inspection images. They conducted an in-depth evaluation of various deep-learning models to determine whether AI could achieve human-level accuracy in analyzing ultrasonic images for defect identification. The study provided detailed benchmarking comparisons, examining aspects such as defect-detection accuracy, model complexity, memory requirements, and inference time. Their findings offered critical insights into the effectiveness of advanced AI models in UT image analysis, providing practitioners with an objective framework to evaluate different approaches and outcomes. Additionally, the authors highlighted the importance of both model-driven and data-driven approaches in NDE data analysis, stressing the significance of computational methods for improving NDE data understanding. Their work contributes significantly to the field by providing an open-access dataset and benchmarking results for deep-learning models, facilitating future research and development in automatic ultrasonic inspection image interpretation for NDE. Figure 29 provides a schematic diagram of the proposed UT combined with the ML approach, illustrating how signal processing and model-interpretation techniques are integrated to optimize feature selection and enhance model transparency for defect characterization and prediction [211].

Recent advancements in AM, specifically FFF, necessitate precise defect detection and characterization to ensure component integrity and performance [151]. NDT methods, including UT, radiography, and thermography, have been widely employed to detect defects in manufactured components. However, these methods often face limitations in resolution and the ability to characterize multi-scale defects intrinsic to FFF processes. The integration of ML into NDT offers a transformative approach to address these challenges. ML-enabled NDT leverages vast datasets and sophisticated algorithms to enhance defect-detection accuracy, automate the characterization process, and provide predictive maintenance insights. By employing advanced techniques such as CNNs and reinforcement learning, ML-enabled systems can process complex patterns and anomalies in real time, surpassing the capabilities of traditional methods [151]. ML algorithms have proven effective in enhancing the sensitivity and accuracy of defect characterization in FFF, improving quality-control processes [214]. Roh et al. [215] investigated the use of ML for the NDT of impact damage in FRTPCs for safety and maintenance. They explored combining self-sensing techniques using carbon fiber with ML for NDE, showcasing the potential of ML in designing smart FRTPCs. The study introduced a novel algorithm for structural health monitoring using an artificial neural network (ANN). The ML models developed do not rely on theoretical models for variables like maximum impact energy, impact force, or fiber type. The ANN-based algorithms effectively localize and assess sensitivity without needing an electromechanical model, improving the accuracy and sensing capabilities of large-scale FRTPCs.

Silva et al. [43] reviewed the intricate challenges associated with detecting and characterizing multi-scale defects, particularly those less than 100 µm, across diverse engineering materials. They highlighted the critical importance of precise defect identification in maintaining the structural integrity of crucial components in high-value applications. The study examined the limitations of traditional NDT techniques in addressing small-scale defects in metallic components, polymeric materials, and composites. It categorized exemplary NDT solutions into stand-alone techniques, hybrid methodologies, algorithms combined with NDT techniques, and ML-aided NDT, showcasing notable applications within each category. For example, standalone methods like time-of-flight diffraction at 7.5 MHz and Thermoreflectance Thermal Imaging (TTI) proved effective in detecting early damage and submicron defects. Hybrid techniques, such as combining CT with computed laminography, offered improved resolution while pairing NDT methods with advanced digital algorithms—like Ultrasonic Phased Array with the Total Focusing Method-boosted sensitivity. Additionally, machine-learning-enhanced NDT techniques, utilizing algorithms such as deep learning and CNN, significantly improved the analysis of complex data. The study highlighted the need for further case studies, particularly those focused on manufacturing and in-service defects, to improve the reliability and effectiveness of these innovative NDT approaches, contributing to enhanced safety and sustainability in high-value engineering systems. Table 15 showcases recent research articles on ML-enabled NDT techniques for defect detection in various composites.

**Figure 30:**
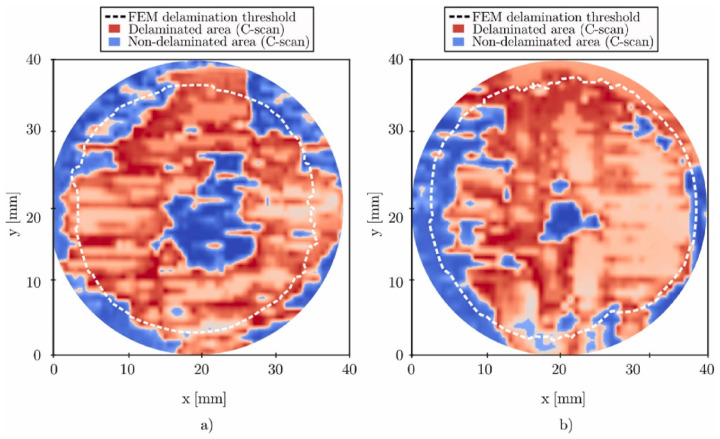
Comparison of delamination areas obtained experimentally through C-Scan and predicted by the FEM model for (**a**) low void content and (**b**) high void content. Reproduced with permission from [54], © 2023 Elsevier.

Mendikute et al. [54] explored the potential of machine-learning-based surrogate models to forecast low-velocity impact behavior, considering void content and distribution within resin-transfer molding. Due to challenges in obtaining reliable experimental datasets, they developed a finite-element model to generate synthetic data (Figure 30). They determined the best hyperparameters for the random-forest (RF) model using grid search and discovered that a multi-output regression model, capable of predicting force-time, displacement-time, and energy-time curves, delivered highly accurate results (R^2^ > 0.995) with a swift processing time of just 5 s per sample. This makes it an effective tool for real-time monitoring of structural performance.

**Figure 31:**
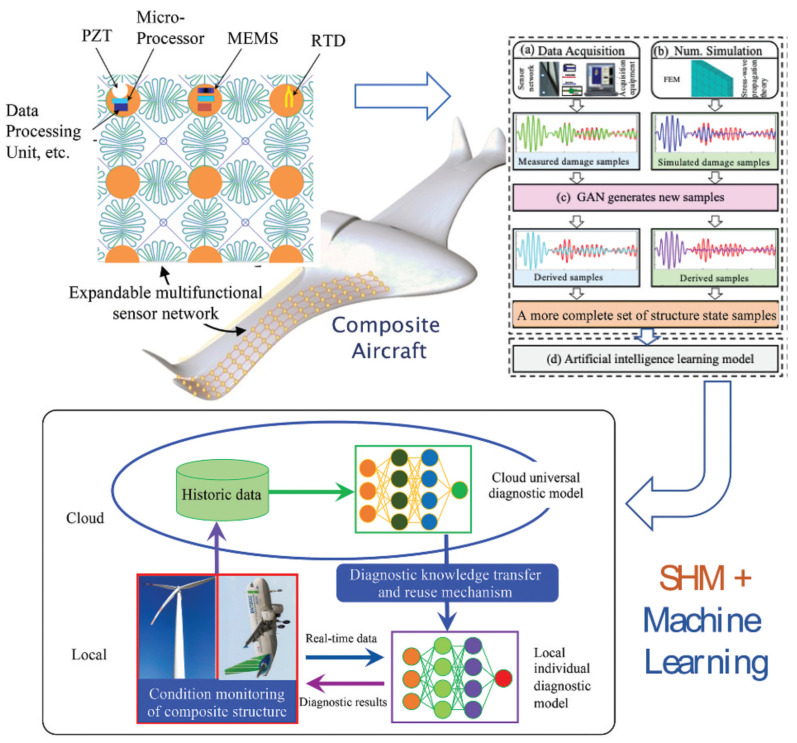
Integrating advanced sensing, numerical simulations, ML, and SHM for characterizing structural damage [121]. Reproduced with permission under CC BY 4.0, © 2022 Taylor and Francis.

Qing et al. [121] undertook a meticulous exploration of composite materials, renowned for their exceptional mechanical properties. This in-depth study sought to tackle the complex challenges related to evaluating the integrity and durability of these materials, considering their unique properties and the intricate interactions between load and environmental conditions. Recognizing the limitations posed by conventional NDT methods, the study introduced SHM as a promising solution, strategically leveraging ML to overcome these challenges. The integration of these technologies with ML marked a paradigm shift in the assessment of structural damage, emphasizing the significance of comprehensive data acquisition and intelligent identification of composite delamination defects. This integration not only promised heightened precision in damage assessment but also opened avenues for a more insightful understanding of the factors influencing composite material behavior. The findings underscored the substantial potential of ML-based SHM for future advancements, ensuring safety and reliability across diverse industries. This research demonstrated that the fusion of ML and SHM could herald a paradigmatic change in damage monitoring, offering a more sophisticated and accurate approach compared to traditional methodologies (Figure 31). This understanding is particularly significant in sectors such as construction and aerospace, where composite materials hold a significant role.

Within the domain of SHM, the application of ML unfolds through two pivotal steps [121]. The first step integrates advanced sensing technologies with numerical simulation techniques to gather monitoring data, enabling effective characterization of structural damage. This foundational step ensures a robust data-driven approach to damage assessment, with advanced sensing technology providing a high-fidelity representation of the structural state, and numerical simulation methods adding a layer of predictive capability for a proactive approach to damage monitoring. Subsequently, the second step employs ML methods to analyze the acquired monitoring data, facilitating an intelligent diagnosis of structural damage. This stage is instrumental in extracting meaningful insights from the wealth of data gathered in the initial step. ML algorithms excel in discerning patterns and hidden correlations within complex datasets, contributing to a better understanding of structural health. The utilization of ML not only enhances the efficiency of damage diagnosis but also contributes to the overall predictive maintenance strategy, enabling timely interventions to prevent catastrophic failures. To visually complement and enhance the understanding of these processes, Figure 31 provides a graphical representation of the intricate process of monitoring damage in composite structures using ML, drawing insights, and methodologies [121]. The AM of FRTPCs presents numerous advantages, including enhanced mechanical properties and the potential for complex geometries. However, the process is not without its challenges, particularly regarding defects induced by FFF. These defects span multiple scales and significantly impact the performance and reliability of FRTPCs. The review conducted on this topic identifies several research gaps that need addressing to advance the field. Table 16 below summarizes these gaps and suggests potential future research directions.

## 4. Conclusions and Future Perspective

In conclusion, this review provides a comprehensive analysis of multiscale defects in FRTPCs produced via FFF, emphasizing the impact of process parameters and the complexities involved in managing these defects. It covers fundamental components, including various FRTPC materials (such as S- and C-FRTPCs), the classification and formation of AM defects, and both conventional and advanced NDT methods for identifying multiscale FFF-induced defects. Moreover, the review addressed sophisticated techniques for characterizing these defects and implementing advanced defect-detection methodologies, along with state-of-the-art SHM techniques integrated with ML algorithms. The incorporation of hybrid methodologies that blend NDT and ML offers a comprehensive understanding of FFF-induced multiscale defects in FRTPCs. Key focal points that emerge from this review are as follows:

- A systematic categorization of FFF-induced defects based on morphology, location, and size can provide a suitable framework to evaluate their effects on long-term mechanical, thermal, and environmental durability.
- The advancement of NDT techniques through the integration of the self-sensing capability of FRTPC materials and high-resolution imaging modes offers the promise of enhancing defect-detection precision and reliability.
- The integration of novel ML algorithms into multiscale defect detection and characterization processes offers transformative potential, providing opportunities for improved sensitivity and real-time monitoring capabilities.

Future research must prioritize several aspects. First, advancements in ML-assisted NDT necessitate the careful refinement of ML models for defect detection and characterization, with a particular emphasis on real-time applications and improved accuracy. Second, the exploration of synergies between NDT techniques and ML algorithms is imperative to develop hybrid methodologies, facilitating comprehensive defect assessment. Third, strengthening collaboration between academia and industry is vital to harnessing the full potential of ML-assisted AM, thereby driving innovation in defect management and quality control. Lastly, investigating self-reporting FRTPCs by analyzing their thermal, electrical, and self-sensing behaviors and integrating them with ML and advanced NDT techniques can advance SHM and multifunctional performance of FFF-fabricated FRTPCs for aerostructural applications. These initiatives can collectively support the development of next-generation defect detection and quality-control systems in FRTPC fabrication, ensuring reliability and performance in critical or high-performance applications.

## Figures and Tables

**Table 4:** Classification of FFF-induced defects [104].

1. Size Range	Examples	2. Spatial Topology	Examples
**Centimeteric**	Shrinkage, warping, layer shifting	0-dimensional	Voids
**Millimetric**	Gaps, stringing, over/under-extrusion	Three dimensional	Stringing
**Micrometric**	Cracks, blobs, voids	Two dimensional	Delamination, banding
**Nanometric**	Molecular cleavage	Three dimensional	Warping, curling
**Angstrom**		Four dimensional	Cracks, defect propagation
**3. Nature of Occurrence**	**Examples**	**4. Location**	**Examples**
**Material Abnormality**	Voids, blobs, stringing, gaps, over/under-extrusion	Surface	Stringing, delamination, banding, warping, curling
**Deformation**	Layer misalignment, curling, shrinkage, cracks, delamination, warping	Internal	Cracks, voids, over/under-extrusion, molecular cleavage
**Chemical Bond Cleavage**	Molecular cleavage	Combined	Shrinkage, layer shifting, defect propagation

**Table 5:** Characterization techniques, advantages, limitations/challenges, and future opportunities in defect-location analysis of FRTPCs in AM processes [43].

Technique	Description	Advantages	Limitations	Challenges	Future Opportunities
**Optical Microscopy**	Visualizes surface features with high spatial resolution.	High spatial resolution. Surface defect detection.	Limited penetration depth. Time-consuming analysis. Sample preparation alters defect distribution.	Subsurface Defects: Detection and characterization of defects beneath the surface domain.	Advanced Characterization Techniques: Development of 3D imaging and multi-modal approaches for comprehensive defect analysis.
**Electron Microscopy**	Provides detailed imaging of surface and internal structures.	High spatial resolution. Surface- and internal-defect analysis.	Vacuum requirements. Specialized sample preparation. Potential alteration of defect morphology.	Effect on Structural Integrity: Implications of defect location for component strength and performance.	Innovative Inspection Methods: Integration of advanced sensing technologies for enhanced defect detection.
**Acoustic Microscopy**	Uses sound waves to detect subsurface defects non-destructively.	Non-destructive subsurface-defect detection.	Limited penetration depth. Requires sophisticated signal processing.	Impact on Performance: Effects of defects within the material thickness on mechanical properties.	Data Analytics and ML: Utilization of ML algorithms for automated defect detection and classification.
**Electromagnetic Techniques**	Utilizes electromagnetic fields for defect detection.	Potential for non-destructive defect detection.	Effectiveness may be limited by material properties. Requires specialized equipment and expertise.	Quality-Assurance Challenges: Limitations of conventional techniques for defect detection and characterization.	Innovative sensor design and material optimization for enhanced defect-detection capabilities.

**Table 7:** Advanced NDT techniques with micro- and nano-scale spatial resolution, highlighting material compatibility, applications, spatial resolution, and their advantages [43]. Adapted with permission under CC BY 4.0, © 2023 Elsevier.

Technique	Materials	Spatial Resolution	Applications	Advantages	Limitations
X-ray Computed Tomography (CT)	Composites, metals, and wood	~1 µm	Pores, voids, and cracks in complex geometries	Three-dimensional analysis and high precision	Expensive and limited to small samples
X-ray Computed Laminography (CL)	Metals and polymers	~10 µm	Cracks and voids in thin components	Suitable for electronics	Limited field of view
Micro-Laser Line Triangulation (Micro-LLT)	Steel and polymers	~100 µm	Micro porosities and surface cracks	High sensitivity	Limited to flat surfaces
Thermal Tomography Imaging (TTI)	Metals and plastics	~1 µm	Hotspots in electronic components	Non-destructive	Limited depth
Digital Holography (DH)	Polymers and glass	~10 µm	Micro fibers and scratches	High-resolution imaging	Sensitive to vibrations
Ultrasonic Phased Array (PA)	Steel, aluminum, and titanium	~100 µm	Micro cracks and corrosion detection	Real-time high resolution	Requires contact, coupling agents
**Laser Ultrasonics (LUT)**	Aluminum and steels	~10 µm	Internal defects under high temperatures	Non-contact, high resolution	Complex setup
Electromagnetic Acoustic Transducers (EMAT)	Steel and aluminum	~100 µm	Deep flaws in thick structures	No couplant needed	Limited to conductive materials
Ground Penetrating Radar (GPR)	Concrete, asphalt	~1 mm	Voids and rebar positioning	Effective for large areas	Limited resolution for fine cracks

**Table 8:** Overview of self-reporting mechanochromic composites for SHM [139,170,172,174,175,176,177].

Feature	Description
Type of Material	Self-reporting mechanochromic composites
Functionality	Provides feedback on structural integrity through visual signals
Application	Suitable for SHM across various engineering structures, such as concrete, steel, asphalt, and composite materials
Advantages Over Other SHM Techniques	(a) Monitors the system in real time during operation (rather than post-operation) (b) Operates wirelessly without the need for data-acquisition systems
Potential Applications of Self-Reporting Materials	FRTPCs used as structural or reinforcing materials can be designed for both strengthening and SHM, eliminating the need for additional sensors Glass fibers functionalized with fluorescent proteins embedded in epoxy can serve as load-bearing elements while also indicating impact-induced delamination
Innovation Opportunities	The use of self-reporting materials offers novel possibilities and designs for replacing traditional sensors

**Table 9:** Comparative evaluation of four piezoresistive-sensing methods according to the selected strategy [118,192].

Piezoresistive-Sensing Approach	Sensitivity	Ease of Manufacturing	Performance Spectrum	Limitation
Self-sensing in carbon-based FRTPCs	Fixed sensitivity	Ranges from simple to complex	Detects fiber-dominated failure modes only	May not detect matrix-dominated failures
Piezoresistive matrices	Partially tailorable	Complex dispersion processes	Detects matrix-dominated failure modes only	Fiber-dominated failures may go unnoticed
Surface-deposited/mounted sensors	Adjustable	Simple to complex	Strain detection only	Inability to detect multiple failure modes
Embedded filaments/yarns	Tailorable	Simple	Detects both matrix and fiber-dominated failures; capable of cure monitoring	-
Hybrid piezoresistive composites	Highly tailorable	Complex	Detects both matrix and fiber-dominated failure modes; improved sensitivity	Higher cost and increased manufacturing complexity
CNT-reinforced sensing layers	Moderately tailorable	Complex dispersions required	High sensitivity to strain and microcracks in matrix	Requires precise dispersion; can be affected by environmental conditions
Multifunctional piezoresistive coatings	Tailorable within limits	Moderate	Capable of detecting surface strain, delamination, and small cracks	Limited durability under cyclic loading conditions

**Table 10:** Different sensing methods and related properties to detect defects in FRTPCs [191,193].

Sensing Method	Sensitivity	Manufacturing Convenience	Performance Spectrum	Limitation	Additional Information
Carbon Fiber Composites with Self-Sensing	Non-customizable	Ranges from straightforward to intricate	Detects only fiber-dominated failure modes	Possible oversight of matrix-dominated failures	Offers real-time feedback on composite integrity. Enhanced tailoring may require advanced fabrication techniques.
Tailorable Piezoresistive Matrices	Adjustable within limits	Complex dispersions	Detects only matrix-dominated failure modes	Potential oversight of fiber-dominated failures	Provides adaptability for specific application requirements within defined limits. Complex dispersions offer a wide range of customization.
Surface-Deposited/Mounted Sensors	Customizable	Ranges from simple to complex	Limited to strain sensing	Inability to correlate responses with various failure modes	Customization extends to sensor placement on the surface, facilitating varied strain measurements. Correlating responses with failure modes may require additional analysis.
Embedded Filaments/Yarns	Tailorable	Convenient manufacturing	Detects both matrix and fiber failures, including cure monitoring	Limited spatial resolution; may struggle with intricate structural configurations or localized damage	Allows for tailoring based on application needs. Enables comprehensive monitoring of multiple failure modes, including curing processes.

**Table 11:** Advantages and disadvantages of primary characterization techniques for void characterization. Adapted with permission from [42], © 2019, SAGE Publications.

Characterization Technique	Measurable Characteristic(s)	Advantages	Disadvantages
Density Measurement	Void content	Simple, cost-effective, and fast	Accuracy relies on input properties, destructive, requires density input, and does not provide detailed void characteristics
Optical Microscopy	Void content, size, 2D shape, and location/distribution	Quick, affordable, and provides 2D morphological details	Biased by section and location, requires multiple analyses, and destructive
Ultrasonic Inspection	Void content, planar size, and location/distribution	Precise, non-destructive, suitable for in-service inspections, and portable	Requires coupling agents, limited to flat/smooth surfaces, slow process, and has limited morphological data
Micro-CT Scanning	Void content, size, 3D shapes, and location/distribution	Highly accurate, offers comprehensive 3D analysis, and minimally destructive	Requires small samples, location-biased, time-consuming, and expensive
Thermography	Surface and subsurface voids and heat distribution	Non-destructive, capable of real-time monitoring, and suitable for large areas	Limited depth resolution, less effective for thick materials, and may require multiple imaging angles
X-ray Radiography	Void content, size, and planar distribution	Non-destructive, effective for internal voids, and suitable for various materials	Limited 3D analysis, may require hazardous X-ray sources, and expensive equipment
Acoustic Emission	Crack initiation and void evolution	Real-time monitoring, non-destructive, and capable of detecting internal damage progression	Complex setup, prone to background noise interference, and requires skilled operators for data interpretation

**Table 12:** Comparative analysis of 2D and 3D imaging in defect characterization and applications [118].

Aspect	Two-Dimensional (2D) Imaging	Three-Dimensional (3D) Imaging
Spatial Information	Limited spatial details and provides a flat representation	Comprehensive exploration of volume, shape, spatial distribution, and depth
Defect Identification	Detects defects but lacks precision in pinpointing	Identifies, precisely localizes, and explains the origin of defects
Structural Complexity Analysis	Struggles with intricate structures and has limited depth perception	Effectively analyzes complex structures, capturing intricate details
Manufacturing Optimization	Offers insights but is limited in guiding precise modifications	Facilitates precise modifications for optimization and efficiency
Material Composition Analysis	Limited capability to analyze material composition	Enables detailed analysis of the material composition, aiding optimization
Engineering Decision-Making	Provides basic information for decision-making	Empowers informed decision-making, crucial for aerospace engineering
In Situ Applications	Challenging for in situ analysis due to limited depth	Facilitates in situ analysis, crucial for real-time monitoring and adjustments
Research and Development	Limited insights for innovative research	Fuels innovative research by providing a detailed understanding of structures
Inhomogeneity	Confirms flaws but lacks depth	Identifies, localizes, and explains flaw origins
Practical Impact	Confirms issues with limited refinement	Facilitates precise modifications for optimization

**Table 13:** Imaging techniques and scale in materials characterization of AM defects [118,198].

Two-Dimensional (2D) Imaging Techniques	Remarks	Three-Dimensional (3D) Imaging Techniques	Remarks
Optical Microscopy: Micro to Meso (1 μm–1 mm)	Provides detailed 2D images of material surfaces and structures	X-ray Computed Tomography (XCT): Micro to Macro (10 μm–1 cm)	Provides detailed 3D images of internal structures and defects
SEM: Nano to Micro (1 nm–1 μm)	Captures high-resolution 2D images of material surfaces using electron beams	Micro-CT: Micro to Macro (1 μm–1 cm)	High-resolution 3D imaging, particularly valuable for materials science research
X-ray Radiography: Nano to Macro (1 nm–1 m)	Offers 2D images, useful for identifying internal structures and defects	Magnetic Resonance Imaging (MRI): Meso to Macro (1 mm–1 cm)	Provides 3D imaging with excellent soft tissue contrast for certain material studies
Infrared (IR) Imaging: Micro to Macro (1 μm–1 m)	Visualizes temperature distribution in 2D, aiding in material characterization	Three-dimensional Scanning Electron Microscopy (3D SEM): Nano to Micro (1 nm–1 μm)	Captures topographical and structural information in 3D
Ultrasonic Testing: Meso to Macro (1 mm–1 m)	Utilizes 2D ultrasound images for NDT of materials	Atomic Force Microscopy (AFM): Nano to Micro (1 nm–1 μm)	Creates 3D images at the nanoscale, revealing surface topography
X-ray Fluorescence (XRF): Nano to Macro (1 nm–1 m)	Analyzes material composition through 2D images of emitted X-rays	Three-dimensional X-ray Tomography: Micro to Macro (1 μm–1 cm)	Utilizes X-rays for detailed 3D reconstructions of material structures
Thin-Section Microanalysis; Micro to Meso (1 μm–1 mm)	Produces 2D images of thinly sliced material sections for detailed analysis	Three-dimensional Ultrasound Imaging: Meso to Macro (1 mm–1 m)	Provides volumetric images for non-destructive evaluation of materials

**Table 14:** Analysis of defects in FFF-printed CFRTPCs and their control strategies.

Defect Type	Root Cause	Control Strategies	Impact on Performance	Monitoring Techniques
Inter-Layer Voids	Insufficient fusion due to low temperature, nozzle misalignment, or contamination	Optimize nozzle temperature, laser-assisted bonding, and post-process annealing	Reduced shear strength, increased delamination, and lower fatigue resistance	Micro CT, SEM, acoustic emission, and ultrasonic inspection
Intra-Layer Voids	Gas entrapment or uneven cooling during material flow	Ultrasonic vibration, controlled cooling rates, and temperature regulation	Decreased tensile and compressive strength and crack initiation	Thermography, micro-CT, acoustic emission, and ultrasound NDT
Fiber Pullout	Weak fiber–matrix bonding under mechanical loads	Surface treatment of fibers, using coupling agents, and optimize fiber alignment	Loss of stiffness, poor load transfer, reduced fatigue life	SEM, acoustic emission, and micro-CT
Fiber Breakage	Excessive stress or poor toolpath causing shear on fibers	Reduce print speed, optimize toolpaths, and improve fiber impregnation	Reduced tensile and flexural strength and brittle fracture	Tensile testing, micro-CT, and high-speed cameras
Fiber Misalignment	Misaligned fibers during extrusion or printing of curved regions	Guided extrusion, AI-based YOLOv8 monitoring, and optimizing print orientation	Reduced load-bearing efficiency and early fatigue failure	Micro CT, visual inspection, and AI-based in situ monitoring
Matrix Cracking	Thermal stress, mechanical loading, or environmental degradation	Thermal annealing, matrix tougheners, and control ambient conditions	Loss of toughness, crack propagation, and early structural failure	Thermography, acoustic emission, and micro-CT
Porosity	Non-uniform material flow, gas entrapment, or unstable extrusion	Feedback-controlled flow, filament-mixing optimization, and temperature stability	Increased anisotropy, reduced mechanical properties, and premature failure	Density analysis, ultrasound NDT, and X-ray CT
Surface Voids/Stair-Stepping	Layer-wise surface roughness, especially on curved surfaces	Use finer layer heights, chemical smoothing agents, and laser polishing	Poor fatigue life, dimensional inaccuracies, and reduced aesthetic quality	Visual inspection, optical profilometry, and white light interferometry
Delamination	Contamination or improper alignment, causing poor inter-layer bonding	Laser-assisted bonding, alignment optimization, and real-time feedback systems	Complete failure under shear and compromised structural integrity	Acoustic emission, micro-CT, and thermography
Warping and Residual Stress	Uneven cooling cycles, thermal gradients, or material shrinkage	Preheating and controlled cooling, fiber reinforcement, and thermal annealing	Dimensional instability, stress concentration, and crack initiation	DIC, thermography, and micro-CT
Contamination Defects	Dust, moisture, or foreign particles in the printing process	Maintain a clean environment, pre-treat materials, and store in sealed containers	Increased porosity, weakened bonding, and reduced mechanical strength	Visual inspection, ultrasonic NDT, and X-ray radiography

**Table 15:** Latest articles on ML-enabled NDT techniques for defect detection in various composites and their applications.

ML Algorithm	NDT Techniques	Defects	Materials/Applications	Ref
CNN	X-ray microtomography	Matrix damage at both the micro and mesoscale levels	Advanced aerospace-grade composite laminate	[216]
(SVM), LVQ, MLP	UT	Classification of matrix cracking	Glass/epoxy cross-ply laminated composites	[217]
RBF-SVM, AEC, HMM, RF	UT	Anomaly detection	Sensor networks for monitoring aluminum and CFRTPC plates used in aerospace and automotive sectors	[218]
SVM and Decision Trees	UT	Defect size	Offline evaluation of fiber metal laminates in A380 aircraft structures using NDT techniques for quality assurance	[219]
K-Means Clustering	Microwave NDT	Defect location and size	GFRTPC for aerospace applications	[38,220]
Mixed algorithms (linear regression, SVM, and Gaussian regression)	Thermography	Defect size	PLA and PA-12	[221]
Mixed algorithms	Thermography, AE, and UT	Delamination, subsurface defect size and depths	CFRTPC	[222,223]

**Table 16:** Identified research gaps (RGs) in the study of AM defects in FRTPCs.

Research Gap ID	Research Gap Description	Current Understanding	Potential Impact	Future Research Direction
RG1	A thorough understanding of multi-scale defects and their combined effect on the mechanical properties of FRTPCs	Studies mainly focus on individual defect scales and defect orientation effects are not well-studied	Improving mechanical properties through defect control and optimization	Investigate the synergistic impacts of multi-scale defects on materials performance through experimental and computational techniques
RG2	Development of advanced NDT methods integrated with ML algorithms for precise defect detection and analysis	Basic NDT methods are commonly used, and few studies integrate NDT and ML for this purpose	Enhanced accuracy in defect detection and characterization	Develop and validate hybrid NDT–ML approaches for comprehensive defect analysis, focusing on real-time applications
RG3	Detailed exploration of the formation mechanisms and mitigation strategies for small-scale (below 100 μm) and FFF-induced defects	Formation mechanisms are partially understood, and current mitigation strategies are not highly effective	Better prediction, control, and reduction of small-scale and FFF-induced defects	Conduct in-depth studies on the formation processes and develop new strategies for mitigating these defects
RG4	Standardization and classification of FFF-induced defects, including the impact of defect orientation on FRTPC properties	Various classifications exist but are not unified and defect orientation effects are understudied	Enhanced defect identification, classification, and understanding of their impact on properties	Develop a standardized classification framework and study the influence of defect orientation on FRTPC properties
RG5	Long-term performance analysis of FRTPCs considering the presence of AM defects	Short-term performance is often studied and basic SHM techniques are currently used	Improved long-term reliability and performance of FRTPCs	Investigate the long-term effects of AM defects on FRTPC performance through accelerated testing and life-cycle analysis

## Abbreviations

List of acronyms for quick reference.

**Acronym**	**Full Form**
AM	Additive Manufacturing
CFRTPC	Continuous Fiber-Reinforced Thermoplastic Composite
FFF	Fused Filament Fabrication
FRP	Fiber-Reinforced Polymer
GFRP	Glass Fiber-Reinforced Polymer
CFRP	Carbon Fiber-Reinforced Polymer
FRTPC	Fiber-Reinforced Thermoplastic Composite
PEEK	Polyether Ether Ketone
GnP	Graphite Nanoplatelets
CNT	Carbon Nanotube
MXene	Transition Metal Carbide or Nitride Materials
rGO	Reduced Graphene Oxide
SHM	Structural Health Monitoring
NDT	Non-Destructive Testing
NDE	Non-Destructive Evaluation
PAUT	Phased Array Ultrasonic Testing
UT	Ultrasonic Testing
CT	Computed Tomography
XCT	X-ray Computed Tomography
IRT	Infrared Thermography
AE	Acoustic Emission
ECT	Eddy-Current Testing
DR	Digital Radiography
GWT	Guided-Wave Testing
LUT	Laser Ultrasonics
THz	Terahertz Imaging
CL	Computed Laminography
LLT	Laser-Line Triangulation
LST	Laser-Spot Thermography
AUBT	Automated Ultrasonic Backscatter Technique
GWUT	Guided-Wave Ultrasonic Testing
EMAT	Electromagnetic Acoustic Transducer
DL	Deep Learning
ML	Machine Learning
CNN	Convolutional Neural Network
CER	Change Electrical Resistance
EMI	Electromagnetic Interference
PVDF	Polyvinylidene Fluoride
IoT	Internet of Things
